# Supplementary material for: Dense active matter model of motion patterns in confluent cell monolayers
Source: Nat Commun. 2020 Mar 16;11:1405. doi: 10.1038/s41467-020-15164-5 (PMC7075903; doi:10.1038/s41467-020-15164-5)
Supplement: Supplementary file 1 — Supplementary Information [file 41467_2020_15164_MOESM1_ESM.pdf]

Supplementary Information

Dense active matter model of motion patterns in confluent cell monolayers

S. Henkes, K. Kostanjevec, J.M. Collinson, R. Sknepnek, E. Bertin

January 22, 2020

Supplementary Note 1

Normal mode formulation. We present here a more detailed methodological account of the normal modes formalism used. We consider a model system of N self-propelled soft interacting particles with overdamped dynamics, in the jammed state. In the absence of self-propulsion, the particles have an equilibrium position $\mathbf{r}_i^0$, corresponding to a local minimum of the elastic energy. If the interaction potential is linearized around the energy minimum in terms of the displacement $\delta\mathbf{r}_i = \mathbf{r}_i - \mathbf{r}_i^0$, the dynamics is described by the equation

$$\zeta \delta\dot{\mathbf{r}}_i = \zeta v_0 \hat{\mathbf{n}}_i - \sum_j \mathbf{K}_{ij} \cdot \delta\mathbf{r}_j, \tag{1}$$

where the $\mathbf{K}_{ij}$'s are the 2×2 blocks of the $2N \times 2N$ dynamical matrix, $v_0\hat{\mathbf{n}}_i$ is the self-propulsion term with $\hat{\mathbf{n}}_i = \cos\phi_i \mathbf{e}_x + \sin\phi_i \mathbf{e}_y$ (i.e., direction of $\hat{\mathbf{n}}$ is given by the angle ϕ_i with the x axis of a laboratory reference frame) and ζ is the friction coefficient. In the absence of inter-particle alignment, the angle ϕ_i obeys a simple rotational diffusive dynamics with white noise $\eta_i(t)$:

$$\dot{\phi}_i = \eta_i(t), \quad \langle \eta_i(t) \rangle = 0, \quad \langle \eta_i(t)\, \eta_j(t') \rangle = \frac{2}{\tau} \delta_{ij} \delta(t - t'), \tag{2}$$

where we have expressed the inverse rotational diffusion constant as a time scale, $\tau = 1/D_r$. We note that in general, the system is far out of thermodynamic equilibrium and D_r and ζ are not simply related to each other. In the following, we consider the self-propulsion noise as a (vectorial) colored noise, and characterize its statistics as well as the statistics of the displacements $\delta\mathbf{r}_i$. To this aim, we first expand $\delta\mathbf{r}_i$ over the normal modes, i.e., the eigenvectors of the dynamical matrix. Each normal mode is a $2N$-dimensional vector that can be written as a list of N two-dimensional vectors $(\boldsymbol{\xi}_1^\nu, \dots, \boldsymbol{\xi}_N^\nu)$, where the index $\nu = 1, \dots, 2N$ labels the mode; the associated eigenvalue is denoted as λ_ν. This form of the normal modes is useful as it allows the decomposition of $\delta\mathbf{r}_i$ to be written in the simple form

$$\delta\mathbf{r}_i = \sum_{\nu=1}^{2N} a_\nu \boldsymbol{\xi}_i^\nu. \tag{3}$$

Projecting Eq. (1) on the normal modes, we find the uncoupled set of equations

$$\zeta \dot{a}_\nu = -\lambda_\nu a_\nu + \eta_\nu, \quad \text{where} \quad \eta_\nu = v_0 \zeta \sum_{i=1}^{2N} \hat{\mathbf{n}}_i \cdot \boldsymbol{\xi}_i^\nu, \tag{4}$$

is the projection of the self-propulsion force onto the normal mode ν.

Self-propulsion force as a persistent noise. We consider the projection η_ν of the self-propulsion force on normal mode ν as a correlated noise, which we now characterize. Since η_ν is the sum of many statistically independent contributions with bounded moments, using the Central Limit theorem, we can assume its statistics to be Gaussian. It is also clear, by averaging over the realizations of the stochastic angles ϕ_i, that $\langle \eta_\nu(t) \rangle = 0$. We thus simply

need to evaluate the two-time correlation function of $\eta_\nu(t)$. Using the fact that the eigenvectors of the dynamical matrix form an orthonormal basis, we have $\sum_{i=1}^{N} \boldsymbol{\xi}_i^\nu \cdot \boldsymbol{\xi}_i^{\nu'} = \delta_{\nu,\nu'}$. We find

$$\langle \eta_\nu(t)\, \eta_{\nu'}(t') \rangle = C(t-t')\, \delta_{\nu,\nu'} \quad \text{with} \quad C(t-t') = \frac{\zeta^2 v_0^2}{2} \langle \cos[\phi(t) - \phi(t')] \rangle , \tag{5}$$

where $\phi(t)$ obeys the diffusive dynamics of Eq. (2). Note that we have used time translation invariance by assuming that the correlation function depends only on the time difference $t - t'$. We can thus set $t' = 0$ without loss of generality. Solving Eq. (2), the quantity $\Delta\phi = \phi(t) - \phi(0)$ is distributed according to

$$p(\Delta\phi, t) = \frac{1}{\sqrt{4\pi|t|/\tau}}\, \mathrm{e}^{-(\Delta\phi)^2 \frac{\tau}{4|t|}} . \tag{6}$$

One then finds, using Eqs. (5) and (6),

$$C(t) = \frac{\zeta^2 v_0^2}{2} \mathrm{e}^{-|t|/\tau}, \tag{7}$$

i.e., the time correlation of the noise η_ν decays exponentially with the correlation (or persistence) time τ. It is worth emphasizing that the statistical properties of the noise η_ν are independent of the mode ν.

Potential energy spectrum. We now turn to the computation of the average potential energy per mode. Solving Eq. (4) explicitly for a given realization of the noise $\eta_\nu(t)$, one finds

$$a_\nu(t) = a_\nu(0)\, \mathrm{e}^{-\frac{\lambda_\nu}{\zeta} t} + \int_0^t dt' \frac{\eta_\nu(t')}{\zeta}\, \mathrm{e}^{-\frac{\lambda_\nu}{\zeta}(t-t')} . \tag{8}$$

From this expression, one can compute the average value $\langle a_\nu^2(t) \rangle$, leading for $t \to \infty$ to

$$\langle a_\nu^2 \rangle = \frac{\zeta}{\lambda_\nu} \int_0^\infty dv\, \frac{1}{\zeta^2} C(v)\, \mathrm{e}^{-\frac{\lambda_\nu}{\zeta} v} . \tag{9}$$

Using Eq. (7), we obtain

$$\langle a_\nu^2 \rangle = \frac{\zeta}{\lambda_\nu} \int_0^\infty dv\, \frac{v_0^2}{2}\, \mathrm{e}^{-v/\tau}\, \mathrm{e}^{-\frac{\lambda_\nu}{\zeta} v} = \frac{\zeta v_0^2}{2\lambda_\nu} \int_0^\infty dv\, \mathrm{e}^{-\left(\frac{1}{\tau} + \frac{\lambda_\nu}{\zeta}\right) v} = \frac{\zeta v_0^2 \tau}{2\lambda_\nu \left(1 + \frac{\lambda_\nu}{\zeta}\tau\right)} \tag{10}$$

or, in terms of average energy per mode

$$E_\nu = \left\langle \frac{1}{2} \lambda_\nu a_\nu^2 \right\rangle = \frac{\zeta v_0^2 \tau}{4\left(1 + \frac{\lambda_\nu}{\zeta}\tau\right)} . \tag{11}$$

For very short correlation time τ (i.e., large diffusion coefficient D_r), one recovers an effective equipartition of energy over the modes, $E_\nu \approx \frac{\zeta v_0^2 \tau}{4}$ even though the system is out-of-equilibrium. For finite correlation time, this result remains valid in the range of modes ν such that $\tau \ll \zeta \lambda_\nu^{-1}$, if such a range exists. However, for large correlation time τ, that is, as soon as there is a wide range of modes such that $\tau \gg \zeta \lambda_\nu^{-1}$, equipartition is broken, and the energy spectrum is given by $E_\nu \approx \frac{\zeta^2 v_0^2}{4\lambda_\nu}$.

Velocity correlation. Following [3], we consider the velocity-velocity correlation function $\hat{G}(\mathbf{q})$ in Fourier space, where one can express the (discrete) Fourier transform $\mathbf{v}(\mathbf{q})$ as a function of the particles reference positions $\mathbf{r}_i^0$:

$$\hat{G}(\mathbf{q}) = \langle \mathbf{v}(\mathbf{q}) \cdot \mathbf{v}^*(\mathbf{q}) \rangle \quad \text{with} \quad \mathbf{v}(\mathbf{q}) = \frac{1}{N} \sum_{j=1}^{N} \mathrm{e}^{\mathrm{i}\mathbf{q}\cdot\mathbf{r}_j^0} \delta\dot{\mathbf{r}}_j , \tag{12}$$

where the star denotes the complex conjugate. Expanding over the normal modes, one finds

$$\hat{G}(\mathbf{q}) = \sum_{\nu,\nu'} \langle \dot{a}_\nu \dot{a}_{\nu'} \rangle\, \boldsymbol{\xi}_\nu(\mathbf{q}) \cdot \boldsymbol{\xi}_{\nu'}^*(\mathbf{q}) , \quad \text{with} \quad \boldsymbol{\xi}_\nu(\mathbf{q}) = \frac{1}{N} \sum_{j=1}^{N} \mathrm{e}^{\mathrm{i}\mathbf{q}\cdot\mathbf{r}_j^0}\, \boldsymbol{\xi}_j^\nu , \tag{13}$$

where $\boldsymbol{\xi}_\nu(\mathbf{q})$ is the Fourier transform of the vectors $\boldsymbol{\xi}_\nu$. From Eq. (4), the quantity $\langle \dot{a}_\nu \dot{a}_{\nu'} \rangle$ is expressed as

$$\langle \dot{a}_\nu \dot{a}_{\nu'} \rangle = \frac{1}{\zeta^2}\left[\lambda_\nu \lambda_{\nu'} \langle a_\nu a_{\nu'} \rangle - \lambda_\nu \langle a_\nu \eta_{\nu'} \rangle - \lambda_{\nu'} \langle a_{\nu'} \eta_\nu \rangle + \langle \eta_\nu \eta_{\nu'} \rangle\right] = \frac{1}{\zeta^2}\left[\lambda_\nu^2 \left\langle a_\nu^2 \right\rangle - 2\lambda_\nu \langle a_\nu \eta_\nu \rangle + \left\langle \eta_\nu^2 \right\rangle\right] \delta_{\nu,\nu'}, \quad (14)$$

where the last equality is due to the modes being uncorrelated. The cross-correlation is in fact not 0, but crucial:

$$\lim_{t\to\infty} \langle a_\nu(t)\, \eta_\nu(t) \rangle = \frac{1}{\zeta}\int_0^\infty dt' \,\langle \eta_\nu(t')\, \eta_\nu(t) \rangle\, \mathrm{e}^{-\frac{\lambda_\nu}{\zeta}(t-t')} = \frac{1}{\zeta}\int_0^\infty dv \frac{\zeta^2 v_0^2}{2}\, \mathrm{e}^{-v/\tau}\, \mathrm{e}^{-\frac{\lambda_\nu}{\zeta}v} = \frac{\zeta v_0^2}{2}\frac{\tau}{1+\frac{\lambda_\nu}{\zeta}\tau}. \quad (15)$$

To sum up, one has according to Eq. (14)

$$\langle \dot{a}_\nu \dot{a}_{\nu'} \rangle = \left\langle \dot{a}_\nu^2 \right\rangle \delta_{\nu,\nu'}, \quad (16)$$

with

$$\left\langle \dot{a}_\nu^2 \right\rangle = \frac{1}{\zeta^2}\left[\lambda_\nu^2 \left\langle a_\nu^2 \right\rangle - 2\lambda_\nu \langle a_\nu \eta_\nu \rangle + \left\langle \eta_\nu^2 \right\rangle\right]. \quad (17)$$

Further, using Eqs. (5), (7), (10) and (15), one obtains

$$\begin{aligned}\left\langle \dot{a}_\nu^2 \right\rangle &= \frac{1}{\zeta^2}\left[\lambda_\nu^2 \frac{\zeta v_0^2 \tau}{2\lambda_\nu (1+\lambda_\nu\tau/\zeta)} - 2\lambda_\nu \frac{\zeta v_0^2}{2}\frac{\tau}{1+\lambda_\nu\tau/\zeta} + \frac{\zeta^2 v_0^2}{2}\right] \\ &= \frac{v_0^2}{2\zeta^2}\frac{1}{1+\frac{\lambda_\nu}{\zeta}\tau}\left[\lambda_\nu\zeta\tau - 2\lambda_\nu\zeta\tau + \zeta^2\left(1+\frac{\lambda_\nu}{\zeta}\tau\right)\right] \\ &= \frac{v_0^2}{2\left(1+\frac{\lambda_\nu}{\zeta}\tau\right)}. \end{aligned} \quad (18)$$

Combining Eqs. (13), (16) and (18), we derive the final expression for the velocity correlation function:

$$\hat{G}(\mathbf{q}) = \sum_\nu \frac{v_0^2}{2\left(1+\frac{\lambda_\nu \tau}{\zeta}\right)} \left\|\boldsymbol{\xi}_\nu(\mathbf{q})\right\|^2. \quad (19)$$

Note that we can compute the equal-time, spatial mean square velocity through Parseval's theorem as

$$\left\langle |\mathbf{v}|^2 \right\rangle = \frac{1}{N}\sum_{j=1}^N \langle |\delta\dot{\mathbf{r}}_j|^2 \rangle = \sum_{\mathbf{q}} \hat{G}(\mathbf{q}) = \frac{L^2}{(2\pi)^2}\int d^2\mathbf{q} \sum_\nu \frac{v_0^2}{2\left(1+\frac{\lambda_\nu\tau}{\zeta}\right)} \left\|\boldsymbol{\xi}_\nu(\mathbf{q})\right\|^2 . \quad (20)$$

Supplementary Note 2

Continuum elastic formulation. We now turn to the study of the overdamped equations of motion derived from the elastic energy, in the framework of continuum elastic. In two dimensions, the elastic energy of an isotropic elastic solid with bulk modulus B and shear modulus μ can be written as [1, 2]

$$F_{\mathrm{el}} = \frac{1}{2}\int d^2\mathbf{r} \left[B\, \mathrm{Tr}\left(\hat{u}(\mathbf{r})\right)^2 + 2\mu\left(u_{\alpha\beta}(\mathbf{r}) - \frac{1}{2}\mathrm{Tr}\left(\hat{u}(\mathbf{r})\right)\delta_{\alpha\beta}\right)^2\right], \quad (21)$$

where $\hat{u}$ is the strain tensor with components $u_{\alpha\beta} = \frac{1}{2}\left[\partial_\alpha u_\beta + \partial_\beta u_\alpha\right]$ written as spatial derivatives of the components $\alpha, \beta \in \{x, y\}$ of the displacement vectors $\mathbf{u}(\mathbf{r}) = \mathbf{r}'(\mathbf{r}) - \mathbf{r}$ from a reference state $\mathbf{r}$ to the deformed state $\mathbf{r}'(\mathbf{r})$. The stress tensor $\sigma_{\alpha\beta} = \frac{\delta F_{\mathrm{el}}}{\delta u_{\alpha\beta}}$ can then be written as

$$\sigma_{\alpha\beta} = B\delta_{\alpha\beta}u_{\gamma\gamma} + 2\mu\left(u_{\alpha\beta} - \frac{1}{2}\delta_{\alpha\beta}u_{\gamma\gamma}\right), \quad (22)$$

where summation over pairs of repeated indices is assumed. Hence, its divergence is given by

$$\partial_\beta \sigma_{\alpha\beta} = B\partial_\alpha u_{\gamma\gamma} + 2\mu\left(\partial_\beta u_{\alpha\beta} - \frac{1}{2}\partial_\alpha u_{\gamma\gamma}\right).$$

We can then write the overdamped equations of motion for the displacement field

$$\zeta \dot{u}_\alpha = \partial_\beta \sigma_{\alpha\beta} = B\partial_\alpha \partial_\gamma u_\gamma + 2\mu \left(\frac{1}{2}\partial_\beta \left(\partial_\alpha u_\beta + \partial_\beta u_\alpha \right) - \frac{1}{2}\partial_\alpha \partial_\gamma u_\gamma \right).$$

This last equation can be rewritten in vectorial notation as

$$\zeta \dot{\mathbf{u}} = B\nabla \left(\nabla \cdot \mathbf{u} \right) + \mu \Delta \mathbf{u}. \tag{23}$$

In Fourier space, we can write this relation as

$$\zeta \dot{\tilde{\mathbf{u}}} = -\mathbf{D}\left(\mathbf{q} \right) \tilde{\mathbf{u}}, \quad \mathbf{D}\left(\mathbf{q} \right) = \begin{bmatrix} Bq_x^2 + \mu q^2 & Bq_x q_y \\ Bq_y q_x & Bq_y^2 + \mu q^2 \end{bmatrix}, \tag{24}$$

where $\mathbf{D}\left(\mathbf{q} \right)$ is the Fourier space dynamical matrix, and $q^2 = q_x^2 + q_y^2$. The two eigenvalues of the dynamical matrix are

$$\lambda_{\mathrm{L}} = \left(B + \mu \right) q^2, \quad \lambda_{\mathrm{T}} = \mu q^2, \tag{25}$$

with normalized eigenvectors

$$\epsilon_{\mathrm{L}} = \frac{1}{q}\left(q_x, q_y \right) \equiv \hat{\mathbf{q}}, \quad \epsilon_{\mathrm{T}} = \frac{1}{q}\left(q_y, -q_x \right) \equiv \hat{\mathbf{q}}^{\perp}. \tag{26}$$

In other words, for each $\mathbf{q}$, we obtain one longitudinal and one transverse eigenmode, with diffusive equations of motion, where the diffusion coefficients are the two elastic moduli:

$$\begin{aligned} \dot{\tilde{u}}_{\mathrm{L}} &= -D_{\mathrm{L}} q^2 \tilde{u}_{\mathrm{L}}, \quad D_{\mathrm{L}} = B + \mu \\ \dot{\tilde{u}}_{\mathrm{T}} &= -D_{\mathrm{T}} q^2 \tilde{u}_{\mathrm{T}}, \quad D_{\mathrm{T}} = \mu. \end{aligned} \tag{27}$$

Overdamped dynamics with activity. Now including the self-propulsion force, the continuum version of the active equations of motion is given by

$$\zeta \dot{\mathbf{u}} = \zeta v_0 \hat{\mathbf{n}} + \boldsymbol{\nabla} \cdot \hat{\boldsymbol{\sigma}}, \tag{28}$$

where we have included an active force $\mathbf{F}^{\mathrm{act}}(\mathbf{r}, t) = \zeta v_0 \hat{\mathbf{n}}(\mathbf{r}, t)$, whose statistical properties will be discussed below. At this stage, we need a brief aside to properly define our conventions for the Fourier transform. This is particularly important because we wish to compare results from numerical simulations and from continuum theory. Numerical simulations are done in a system of relatively large, but finite linear size L, and with a minimal length scale given by the particle size a, which leads to the use of a discrete space Fourier transform. On the other hand, analytical calculations are made much easier by assuming whenever possible that $L \to \infty$ and $a \to 0$, i.e., using the continuous Fourier transform. For consistency between the two approaches, we use the following space continuous Fourier transform

$$\mathbf{u}(\mathbf{r}, t) = \frac{1}{(2\pi)^2} \int d^2\mathbf{q}\, \tilde{\mathbf{u}}(\mathbf{q}, t)\, e^{-i\mathbf{q}\cdot\mathbf{r}} \tag{29}$$

$$\tilde{\mathbf{u}}(\mathbf{q}, t) = \int d^2\mathbf{r}\, \mathbf{u}(\mathbf{r}, t)\, e^{i\mathbf{q}\cdot\mathbf{r}}. \tag{30}$$

When the finite system and particle sizes need to be taken into account, we discretize the integrals into

$$\frac{1}{(2\pi)^2} \int d^2\mathbf{q} \to \frac{1}{Na^2} \sum_{\mathbf{q}}, \qquad \int d^2\mathbf{r} \to a^2 \sum_{\mathbf{r}}, \tag{31}$$

where $N = L^2/a^2$ is the number of particles, at unity packing fraction. In the sum, $\mathbf{q}$ takes discrete values defined by the geometry of the problem. For instance, for a square lattice of linear size L, $\mathbf{q} = (2\pi m/L, 2\pi n/L)$ where (m, n) are integers satisfying $0 \leq m, n \leq L/a - 1$. From this discretization, we get that the discrete space Fourier transform $\mathbf{u}(\mathbf{q}, t)$ is consistently related to the continuous Fourier transform $\tilde{\mathbf{u}}(\mathbf{q}, t)$ through

$$\tilde{\mathbf{u}}(\mathbf{q}, t) = a^2 \mathbf{u}(\mathbf{q}, t). \tag{32}$$

This relation will be useful for comparison to the results of numerical simulations. In the following, we generically use the tilde notation for continuous Fourier transform, and drop the tilde when dealing with the discrete Fourier transform.

To proceed with the computations in the framework of the continuum theory, we now introduce the space and time Fourier transform

$$\mathbf{u}(\mathbf{r},t) = \frac{1}{(2\pi)^3}\int d^2\mathbf{q}\int d\omega\,\tilde{\mathbf{u}}(\mathbf{q},\omega)\,\mathrm{e}^{-\mathrm{i}\mathbf{q}\cdot\mathbf{r}-\mathrm{i}\omega t} \tag{33}$$

$$\tilde{\mathbf{u}}(\mathbf{q},\omega) = \int d^2\mathbf{r}\int dt\,\mathbf{u}(\mathbf{r},t)\,\mathrm{e}^{\mathrm{i}\mathbf{q}\cdot\mathbf{r}+\mathrm{i}\omega t}. \tag{34}$$

With these definitions, the active equation of motion (28) can be rewritten in Fourier space as

$$-\mathrm{i}\zeta\omega\tilde{\mathbf{u}}(\mathbf{q},\omega) = \tilde{\mathbf{F}}^{\mathrm{act}}(\mathbf{q},\omega) - \mathbf{D}(\mathbf{q})\tilde{\mathbf{u}}(\mathbf{q},\omega) \tag{35}$$

where we have defined the continuous Fourier transform $\tilde{\mathbf{F}}^{\mathrm{act}}(\mathbf{q},\omega)$ of the random active force $\mathbf{F}^{\mathrm{act}}(\mathbf{r},t)$ in Fourier space as

$$\tilde{\mathbf{F}}^{\mathrm{act}}(\mathbf{q},\omega) = \zeta v_0\int d^2\mathbf{r}\int_{-\infty}^{\infty} dt\,\hat{\mathbf{n}}(\mathbf{r},t)\,\mathrm{e}^{\mathrm{i}\mathbf{q}\cdot\mathbf{r}+\mathrm{i}\omega t}. \tag{36}$$

Active noise correlations. To determine the correlation of the active noise, we need to start from a spatially discretized version of the model. For definiteness, we assume a square grid with lattice spacing a. Then for each grid node i we have $\hat{\mathbf{n}}_i = (\cos\phi_i, \sin\phi_i)$ with dynamics $\dot{\phi}_i = \eta_i$, $\langle \eta_i(t)\,\eta_j(t')\rangle = \frac{2}{\tau}\delta_{ij}\delta(t-t')$, and the noise remains spatially uncorrelated. We thus have

$$\langle \hat{\mathbf{n}}_i(t)\cdot\hat{\mathbf{n}}_j(t')\rangle = \delta_{i,j}\,\mathrm{e}^{-|t-t'|/\tau}\,. \tag{37}$$

The exponential time dependence has been obtained using the same reasoning as in Eqs. (5) to (7). In order to take a continuum limit, we replace $\hat{\mathbf{n}}_i$ by a continuous field, and we substitute $\delta_{i,j}$ by its Dirac counterpart, namely

$$\delta_{i,j} \;\to\; a^2\,\delta(\mathbf{r}-\mathbf{r}'). \tag{38}$$

We then have that, in the continuum limit,

$$\langle \hat{\mathbf{n}}(\mathbf{r},t)\cdot\hat{\mathbf{n}}(\mathbf{r}',t')\rangle = a^2\,\delta(\mathbf{r}-\mathbf{r}')\,\mathrm{e}^{-|t-t'|/\tau}\,. \tag{39}$$

In view of Eq. (36), it is clear that $\left\langle \tilde{\mathbf{F}}^{\mathrm{act}}(\mathbf{q},\omega)\right\rangle = 0$, as $\langle\cos\phi\rangle = \langle\sin\phi\rangle = 0$. The second order correlations are simply

$$\left\langle \tilde{\mathbf{F}}^{\mathrm{act}}(\mathbf{q},\omega)\cdot\tilde{\mathbf{F}}^{\mathrm{act}}(\mathbf{q}',\omega')\right\rangle = \zeta^2 v_0^2\int_{-\infty}^{\infty}dt\int_{-\infty}^{\infty}dt'\int d^2\mathbf{r}\int d^2\mathbf{r}'\mathrm{e}^{\mathrm{i}\omega t}\mathrm{e}^{\mathrm{i}\mathbf{q}\cdot\mathbf{r}}\mathrm{e}^{\mathrm{i}\omega' t'}\mathrm{e}^{\mathrm{i}\mathbf{q}'\cdot\mathbf{r}'}\left\langle \hat{\mathbf{n}}(\mathbf{r},t)\cdot\hat{\mathbf{n}}(\mathbf{r}',t')\right\rangle. \tag{40}$$

Using Eqs. (39) and (40), a straightforward calculation then yields

$$\langle \tilde{\mathbf{F}}^{\mathrm{act}}(\mathbf{q},\omega)\cdot\tilde{\mathbf{F}}^{\mathrm{act}}(\mathbf{q}',\omega')\rangle = (2\pi)^3 a^2\zeta^2 v_0^2\,\frac{2\tau}{1+(\tau\omega)^2}\,\delta(\mathbf{q}+\mathbf{q}')\,\delta(\omega+\omega')\,. \tag{41}$$

Note that Eq. (41) is obtained in the continuum formulation, where $\delta(\mathbf{q}+\mathbf{q}')$ is a Dirac delta distribution, which is infinite if one sets $\mathbf{q}' = -\mathbf{q}$. To compare with the numerics, one has to come back to the discrete formulation, corresponding to a finite system size L. The Dirac delta is then replaced by a Kronecker delta according to the substitution rule

$$\delta(\mathbf{q}+\mathbf{q}') \;\to\; \frac{1}{(\Delta q)^2}\,\delta_{\mathbf{q}',-\mathbf{q}} \qquad \text{with}\;\; \Delta q \equiv \frac{2\pi}{L}. \tag{42}$$

We are thus led to define the space-discrete Fourier transform $\mathbf{F}^{\mathrm{act}}(\mathbf{q},\omega) = \tilde{\mathbf{F}}^{\mathrm{act}}(\mathbf{q},\omega)/a^2$ [see Eq. (32)] for discrete wavevectors $\mathbf{q}$ (note that ω remains a continuous variable). The correlation of the discrete Fourier transform $\mathbf{F}^{\mathrm{act}}(\mathbf{q},\omega)$ of the active noise then reads

$$\langle \mathbf{F}^{\mathrm{act}}(\mathbf{q},\omega)\cdot\mathbf{F}^{\mathrm{act}}(-\mathbf{q},\omega')\rangle = 2\pi N\zeta^2 v_0^2\,\frac{2\tau}{1+(\tau\omega)^2}\,\delta(\omega+\omega'), \tag{43}$$

in agreement with Eq. (9) of the main text.

Fourier modes properties. We decompose equation (35) into longitudinal and transverse modes: $\tilde{\mathbf{u}}(\mathbf{q},\omega) = \tilde{u}_{\rm L}(\mathbf{q},\omega)\,\hat{\mathbf{q}} + \tilde{u}_{\rm T}(\mathbf{q},\omega)\,\hat{\mathbf{q}}^{\perp}$ along and perpendicular to the eigenvectors of the dynamical matrix, Eq. (24). We obtain two equations

$$-\,{\rm i}\zeta\omega\tilde{u}_{\rm L}(\mathbf{q},\omega) = \tilde{\mathbf{F}}^{\rm act}(\mathbf{q},\omega)\cdot\hat{\mathbf{q}} - (B+\mu)\,q^2\tilde{u}_{\rm L}(\mathbf{q},\omega)\,,$$
$$-\,{\rm i}\zeta\omega\tilde{u}_{\rm T}(\mathbf{q},\omega) = \tilde{\mathbf{F}}^{\rm act}(\mathbf{q},\omega)\cdot\hat{\mathbf{q}}^{\perp} - \mu q^2\tilde{u}_{\rm T}(\mathbf{q},\omega)\,,$$

with solution

$$\tilde{u}_{\rm L}(\mathbf{q},\omega) = \frac{\tilde{F}^{\rm act}_{\rm L}(\mathbf{q},\omega)}{-{\rm i}\zeta\omega + (B+\mu)\,q^2}, \qquad \tilde{u}_{\rm T}(\mathbf{q},\omega) = \frac{\tilde{F}^{\rm act}_{\rm T}(\mathbf{q},\omega)}{-{\rm i}\zeta\omega + \mu q^2}, \tag{44}$$

where $\tilde{F}^{\rm act}_{\rm L}(\mathbf{q},\omega) = \tilde{\mathbf{F}}^{\rm act}(\mathbf{q},\omega)\cdot\hat{\mathbf{q}}$ and $\tilde{F}^{\rm act}_{\rm T}(\mathbf{q},\omega) = \tilde{\mathbf{F}}^{\rm act}(\mathbf{q},\omega)\cdot\hat{\mathbf{q}}^{\perp}$.

We can use these expressions to obtain velocity correlation functions that can be directly measured in experiments and simulations. As $\tilde{\mathbf{v}}(\mathbf{q},\omega) = -{\rm i}\omega\tilde{\mathbf{u}}(\mathbf{q},\omega)$, we can simply write

$$\begin{aligned}\langle\tilde{\mathbf{v}}(\mathbf{q},\omega)\cdot\tilde{\mathbf{v}}(\mathbf{q}',\omega')\rangle &= \langle\tilde{v}_{\rm L}(\mathbf{q},\omega)\,\tilde{v}_{\rm L}(\mathbf{q}',\omega')\rangle + \langle\tilde{v}_{\rm T}(\mathbf{q},\omega)\,\tilde{v}_{\rm T}(\mathbf{q}',\omega')\rangle \\ &= -\omega\omega'\,\langle\tilde{u}_{\rm L}(\mathbf{q},\omega)\,\tilde{u}_{\rm L}(\mathbf{q}',\omega')\rangle - \omega\omega'\,\langle\tilde{u}_{\rm T}(\mathbf{q},\omega)\,\tilde{u}_{\rm T}(\mathbf{k}',\omega')\rangle\,.\end{aligned}$$

It is easy to show that the longitudinal and transverse components of the active force contribute equally to the correlation, namely

$$\langle\tilde{F}^{\rm act}_{\rm L}(\mathbf{q},\omega)\tilde{F}^{\rm act}_{\rm L}(\mathbf{q}',\omega')\rangle = \langle\tilde{F}^{\rm act}_{\rm T}(\mathbf{q},\omega)\tilde{F}^{\rm act}_{\rm T}(\mathbf{q}',\omega')\rangle = \frac{1}{2}\langle\tilde{\mathbf{F}}^{\rm act}(\mathbf{q},\omega)\cdot\tilde{\mathbf{F}}^{\rm act}(\mathbf{q}',\omega')\rangle\,. \tag{45}$$

Using Eqs. (41), (44) and (45), the correlation functions of the longitudinal and transverse components of the Fourier velocity field are then straightforward to compute, leading to

$$\langle\tilde{v}_{\rm L}(\mathbf{q},\omega)\tilde{v}_{\rm L}(\mathbf{q}',\omega')\rangle = \frac{(2\pi)^3a^2\zeta^2v_0^2\tau\omega^2}{[(B+\mu)^2q^4+\zeta^2\omega^2]\,[1+(\tau\omega)^2]}\,\delta(\mathbf{q}+\mathbf{q}')\,\delta(\omega+\omega') \tag{46}$$

$$\langle\tilde{v}_{\rm T}(\mathbf{q},\omega)\tilde{v}_{\rm T}(\mathbf{q}',\omega')\rangle = \frac{(2\pi)^3a^2\zeta^2v_0^2\tau\omega^2}{[\mu^2q^4+\zeta^2\omega^2]\,[1+(\tau\omega)^2]}\,\delta(\mathbf{q}+\mathbf{q}')\,\delta(\omega+\omega'). \tag{47}$$

Of particular interest is the equal-time Fourier transform of the velocity. In other words, we need to integrate over frequency. E.g., for the longitudinal velocity, we find

$$\begin{aligned}\langle\tilde{v}_{\rm L}(\mathbf{q},t)\,\tilde{v}_{\rm L}(\mathbf{q}',t)\rangle &= \frac{1}{(2\pi)^2}\int_{-\infty}^{\infty}d\omega\int_{-\infty}^{\infty}d\omega' {\rm e}^{-{\rm i}(\omega+\omega')t}\,\langle\tilde{v}_{L}(\mathbf{q},\omega)\,\tilde{v}_{L}(\mathbf{q}',\omega')\rangle \\ &= 2\pi a^2\zeta^2v_0^2\tau\,\delta(\mathbf{q}+\mathbf{q}')\int_{-\infty}^{\infty}d\omega\,\frac{\omega^2}{[(B+\mu)^2q^4+\zeta^2\omega^2]\,[1+(\tau\omega)^2]}.\end{aligned}$$

Using the decomposition

$$\frac{\omega^2}{[(B+\mu)^2q^4+\zeta^2\omega^2]\,[1+(\tau\omega)^2]} = \frac{1}{\mu^2\tau^2q^4-\zeta^2}\left(\frac{\mu^2q^4}{\mu^2q^4+\zeta^2\omega^2} - \frac{1}{1+\tau^2\omega^2}\right) \tag{48}$$

a straightforward integration leads to

$$\langle\tilde{v}_{\rm L}(\mathbf{q},t)\,\tilde{v}_{\rm L}(\mathbf{q}',t)\rangle = \frac{2\pi^2a^2\zeta v_0^2}{(B+\mu)\tau q^2+\zeta}\,\delta(\mathbf{q}+\mathbf{q}'). \tag{49}$$

A similar calculation for the transverse component of the Fourier velocity field yields

$$\langle\tilde{v}_{\rm T}(\mathbf{q},t)\,\tilde{v}_{\rm T}(\mathbf{q}',t)\rangle = \frac{2\pi^2a^2\zeta v_0^2}{\mu\tau q^2+\zeta}\,\delta(\mathbf{q}+\mathbf{q}'). \tag{50}$$

Introducing the longitudinal and transverse characteristic length scales

$$\xi_{\rm L} = \left(\frac{(B+\mu)\tau}{\zeta}\right)^{1/2}, \qquad \xi_{\rm T} = \left(\frac{\mu\tau}{\zeta}\right)^{1/2}, \tag{51}$$

the equal-time (continuous) Fourier velocity correlation can be expressed as

$$\langle \tilde{\mathbf{v}}(\mathbf{q},t)\cdot\tilde{\mathbf{v}}(\mathbf{q}',t)\rangle = 2\pi^2 a^2 v_0^2\left[\frac{1}{1+(\xi_{\mathrm{L}}q)^2}+\frac{1}{1+(\xi_{\mathrm{T}}q)^2}\right]\delta(\mathbf{q}+\mathbf{q}'). \tag{52}$$

The length scales ξ_{L} and ξ_{T} can be interpreted as the longitudinal and transverse correlations lengths that both diverge $\sim \tau^{1/2}$ for $\tau\to\infty$ (i.e., a fully persistent self-propulsion). The existence of those correlation lengths is a direct consequence of activity. In the "passive" limit $\tau\to 0$, these length scales vanish.

It is important to note that Eq. (52) is obtained in the continuum formulation, where $\delta(\mathbf{q}+\mathbf{q}')$ is a Dirac delta distribution. Hence $\langle \tilde{\mathbf{v}}(\mathbf{q},t)\cdot\tilde{\mathbf{v}}(\mathbf{q}',t)\rangle$ is infinite if one sets $\mathbf{q}'=-\mathbf{q}$. To compare with the numerics, one has to come back to the discrete formulation, corresponding to a finite system size L. The Dirac delta is then replaced by a Kronecker delta according to the substitution rule given in Eq. (42). One also needs to replace the continuum Fourier transform $\tilde{\mathbf{v}}(\mathbf{q},t)$ with the discrete one, $\mathbf{v}(\mathbf{q},t)$, according to $\tilde{\mathbf{v}}(\mathbf{q},t)=a^2\mathbf{v}(\mathbf{q},t)$ [see Eq. (32)]. We thus end up with, using $N=L^2/a^2$,

$$\langle \mathbf{v}(\mathbf{q},t)\cdot\mathbf{v}(-\mathbf{q},t)\rangle = N\frac{v_0^2}{2}\left[\frac{1}{1+(\xi_{\mathrm{L}}q)^2}+\frac{1}{1+(\xi_{\mathrm{T}}q)^2}\right], \tag{53}$$

which is precisely Eq. (10) of the main text.

In addition, one can also compute (using integration techniques in the complex plane) the two-time Fourier velocity correlation $\langle \tilde{\mathbf{v}}(\mathbf{q},t)\cdot\tilde{\mathbf{v}}(\mathbf{q}',t')\rangle$. This two-time correlation function is found to decay with the time lag $|t-t'|$ over three different characteristic times, the persistence time τ of the noise and two elastic time scales $\tau_{\mathrm{L}}=\frac{\zeta}{(B+\mu)q^2}$ and $\tau_{\mathrm{T}}=\frac{\zeta}{\mu q^2}$ associated with longitudinal and transverse modes respectively.

Mean-square velocity and velocity autocorrelation function. We conclude by computing the real-space mean-square velocity $\langle|\mathbf{v}(\mathbf{r},t)|^2\rangle$. One has

$$\langle \mathbf{v}(\mathbf{r},t)\cdot\mathbf{v}(\mathbf{r},t)\rangle = \frac{1}{(2\pi)^4}\int d^2\mathbf{q}\int d^2\mathbf{q}'\,\langle \tilde{\mathbf{v}}(\mathbf{q},t)\cdot\tilde{\mathbf{v}}(\mathbf{q}',t)\rangle\,\mathrm{e}^{-\mathrm{i}(\mathbf{q}+\mathbf{q}')\cdot\mathbf{r}}. \tag{54}$$

Using Eq. (52) we get

$$\langle \mathbf{v}(\mathbf{r},t)\cdot\mathbf{v}(\mathbf{r},t)\rangle = \frac{a^2v_0^2}{8\pi^2}\int d^2\mathbf{q}\left[\frac{1}{1+(\xi_{\mathrm{L}}q)^2}+\frac{1}{1+(\xi_{\mathrm{T}}q)^2}\right]. \tag{55}$$

This integral diverges at the upper boundary. This divergence can be regularized if we note that the physical upper limit to this integral is set by the inverse particle size, i.e., by $q_{\mathrm{m}}=\frac{2\pi}{a}$. Therefore, using $\int d^2q = 2\pi\int q\,dq=\pi\int d(q^2)$ when integrating a function of q^2, one obtains

$$\begin{aligned}\left\langle|\mathbf{v}|^2\right\rangle &= \frac{a^2v_0^2}{4\pi}\int_0^{q_{\mathrm{m}}} dq\,q\left[\frac{1}{1+\xi_{\mathrm{L}}^2q^2}+\frac{1}{1+\xi_{\mathrm{T}}^2q^2}\right]\\ &= \frac{v_0^2}{8\pi}\left[\frac{a^2}{\xi_{\mathrm{L}}^2}\log\left(1+\xi_{\mathrm{L}}^2q_{\mathrm{m}}^2\right)+\frac{a^2}{\xi_{\mathrm{T}}^2}\log\left(1+\xi_{\mathrm{T}}^2q_{\mathrm{m}}^2\right)\right].\end{aligned}$$

Note that $\left\langle|\mathbf{v}(\mathbf{r},t)|^2\right\rangle$ is independent of position (and time) and is thus also equal to

$$\left\langle|\mathbf{v}|^2\right\rangle_{\text{space, ensemble}} \equiv \frac{1}{L^2}\int d^2r\left\langle|\mathbf{v}(\mathbf{r},t)|^2\right\rangle. \tag{56}$$

Finally, generalizing the above calculation one can also compute the autocorrelation function of the velocity field, yielding

$$\langle \mathbf{v}(t)\cdot\mathbf{v}(0)\rangle_{\text{space,ensemble}} = \frac{a^2v_0^2\zeta}{4\pi\tau}\int_0^{q_{\mathrm{m}}} dq\,q\left[\frac{(B+\mu)\,q^2\mathrm{e}^{-\frac{B+\mu}{\zeta}q^2t}-\frac{\zeta}{\tau}\mathrm{e}^{-t/\tau}}{(B+\mu)^2q^4-\left(\frac{\zeta}{\tau}\right)^2}+\frac{\mu q^2\mathrm{e}^{-\frac{\mu}{\zeta}q^2t}-\frac{\zeta}{\tau}\mathrm{e}^{-t/\tau}}{\mu^2q^4-\left(\frac{\zeta}{\tau}\right)^2}\right]. \tag{57}$$

Real space expression of the velocity autocorrelation function. We derive here the real space expression for the correlation of velocities of cells separated by r. This is analytically tractable only if the continuum inverse Fourier transform is used, i.e., in the limit of infinite system size. However, this calculation has to be done with care, using the discrete Fourier transform and eventually taking the infinite volume limit to evaluate sums as integrals. Using instead the continuum Fourier transform of the velocity field would lead to difficulties because of the delta function $\delta(\mathbf{q}+\mathbf{q}')$ in Eq. (52). We define the real space correlation function of the velocity field as

$$C_{vv}(\mathbf{r}) = \frac{1}{L^2}\int d^2\mathbf{r}_0 \langle \mathbf{v}(\mathbf{r}_0+\mathbf{r})\cdot\mathbf{v}(\mathbf{r}_0)\rangle \tag{58}$$

as well as its Fourier transform

$$C_{vv}(\mathbf{q}) = \int d^2\mathbf{r}\, C_{vv}(\mathbf{r})\, \mathrm{e}^{\mathrm{i}\mathbf{q}\cdot\mathbf{r}}. \tag{59}$$

Note that the space integration is done on the finite volume L^2, so that the wavevector $\mathbf{q}$ is discretized. A straightforward calculation leads to

$$C_{vv}(\mathbf{q}) = \frac{a^2}{N}\langle|\mathbf{v}(\mathbf{q})|^2\rangle, \tag{60}$$

where $\mathbf{v}(\mathbf{q})$ is the discrete Fourier transform of the velocity field, and $\langle|\mathbf{v}(\mathbf{q})|^2\rangle$ is given in Eq. (53) as well as in Eq. (10) of the main text. From Eq. (60), one can evaluate $C_{vv}(\mathbf{r})$ by computing the inverse Fourier transform of $C_{vv}(\mathbf{q})$. The inverse discrete Fourier transform of $C_{vv}(\mathbf{q})$ can be turned into an integral by taking the limit $L\to\infty$, yielding

$$C_{vv}(\mathbf{r}) = \frac{1}{(2\pi)^2}\int d^2\mathbf{q}\, C_{vv}(\mathbf{q})\, \mathrm{e}^{-\mathrm{i}\mathbf{q}\cdot\mathbf{r}}. \tag{61}$$

Using Eqs. (60) and (53), we obtain

$$C_{vv}(\mathbf{r}) = \frac{a^2 v_0^2}{4\pi}\left[\frac{K_0(r/\xi_\mathrm{L})}{\xi_\mathrm{L}^2} + \frac{K_0(r/\xi_\mathrm{T})}{\xi_\mathrm{T}^2}\right], \tag{62}$$

with $r=|\mathbf{r}|$, and K_0 the modified Bessel function of the second kind. To obtain Eq. (62), we have made use of the following identities involving Bessel functions [4]

$$\frac{1}{2\pi}\int_{-\pi}^{\pi} d\theta\, \mathrm{e}^{\mathrm{i}x\sin\theta} = J_0(x), \qquad \int_0^\infty dx\, \frac{x\, J_0(rx)}{x^2+k^2} = K_0(kr), \tag{63}$$

where J_0 is the Bessel function of the first kind. An asymptotic expansion of Eq. (62) for $r \gg \xi_{\mathrm{L,T}}$ yields

$$C_{vv}(\mathbf{r}) \approx \frac{a^2 v_0^2}{4\pi}\sqrt{\frac{\pi}{2r}}\left(\frac{1}{\xi_\mathrm{L}^{3/2}}\,\mathrm{e}^{-r/\xi_\mathrm{L}} + \frac{1}{\xi_\mathrm{T}^{3/2}}\,\mathrm{e}^{-r/\xi_\mathrm{T}}\right), \tag{64}$$

that is, an exponential decay of $C_{vv}(\mathbf{r})$ at large distances, with algebraic corrections.

Supplementary Note 3

Fitting to experiment and simulations. To compare simulations to our continuum predictions, we need to determine B and μ. As detailed in the Supplementary Note 2, we determine $D(\mathbf{q})$ by Fourier-transforming the dynamical matrix on the $\mathbf{q}$ grid appropriate to the simulations box. The longitudinal and transverse eigenvalues of the resulting 2×2 matrix are then $(B+\mu)q^2$ and μq^2, respectively. In Supplementary Figure 1A, we show the radially $\mathbf{q}$-averaged eigenvalues (dots) as a function of q^2, and the linear fit of the 15 first points we use to extract the moduli.

In Supplementary Figure 1B, we show the Self-Intermediate function as a function of time for the experiments and all three fitted simulations. For the experiment, we numerically integrated the PIV field to obtain approximate trajectories for the regions belonging to each individual PIV arrow at $t=0$. Significant local non-affine motion and distortions emerged, and we stopped before $t=10$ hours and at motions of a couple of cell diameters. The match between experiment and simulation is good for the soft disk simulations; the much slower dynamics of the vertex model is due to its much higher bulk modulus for a given shear modulus at $\bar{p}_0 = 3.6$.

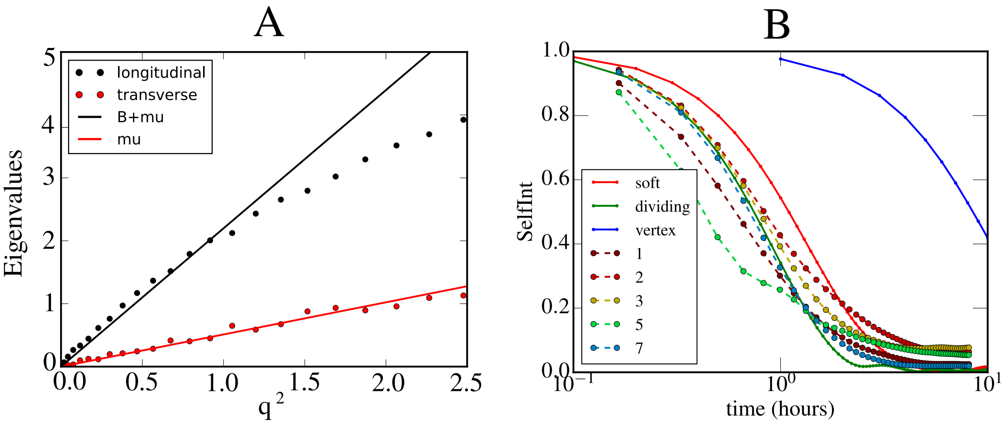

Supplementary Figure 1: A. Determining the elastic moduli for the soft disk model. Shown are the radially averaged longitudinal and transverse eigenmodes of $\mathbf{D}(\mathbf{q})$, determined on the inverse $\mathbf{q}$-lattice appropriate to the simulation box. When plotted against q^2, the longitudinal slope is $B + \mu$, and the transverse slope is μ. Here the static configuration was equilibrated from $T_{\text{eff}} = 0.005$ and $\tau = 200$, but results for other conditions are indistinguishable. B. Velocity autocorrelation function for the experiments, and for the same soft disk and vertex model simulations as in Fig. 4b of the main text. B. Self-Intermediate scattering function for the experiments, and for the same two soft disk simulations and the vertex model simulation as in the main text.

Supplementary Figures

Additional numerical simulation results on the Fourier velocity correlations and their dependence on the persistence time τ are shown in Supplementary Figures 2 and 3. In addition, Supplementary Figure 4 shows the real-space correlation functions for soft disks (left) and the vertex model (right), together with the predictions of Eq. (14) of the main text.

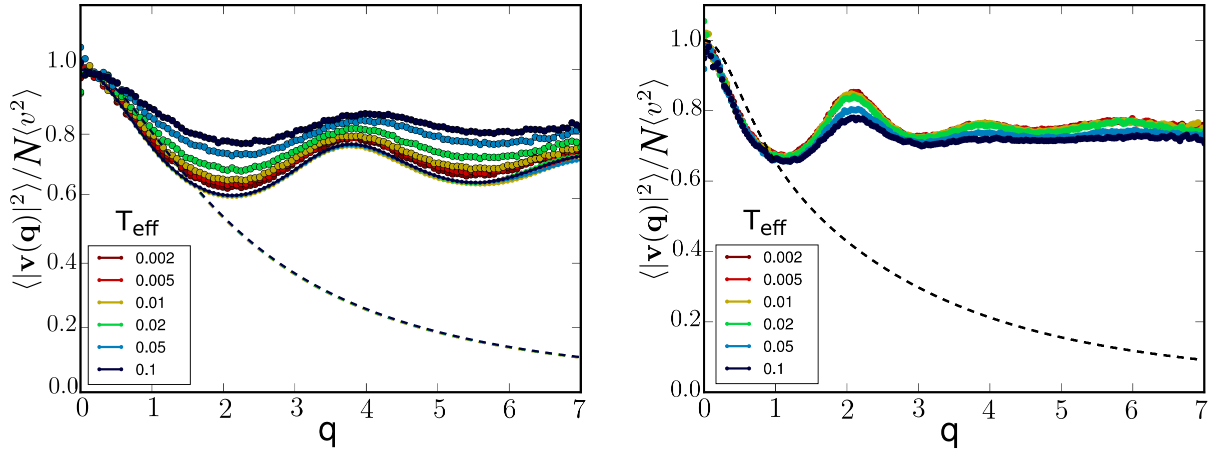

Supplementary Figure 2: In the limit of $\tau \to 0$, we recover the static structure factor $S(q)$. Left: Velocity correlation as a function of T_{eff} for the soft disk system at $\tau = 0.2$, numerically obtained (dots), from the normal modes calculation (lines), and the elastic approximation (dashed line). Right: Same for the vertex model potential, numerical results as dots and elastic approximation as dashed line.

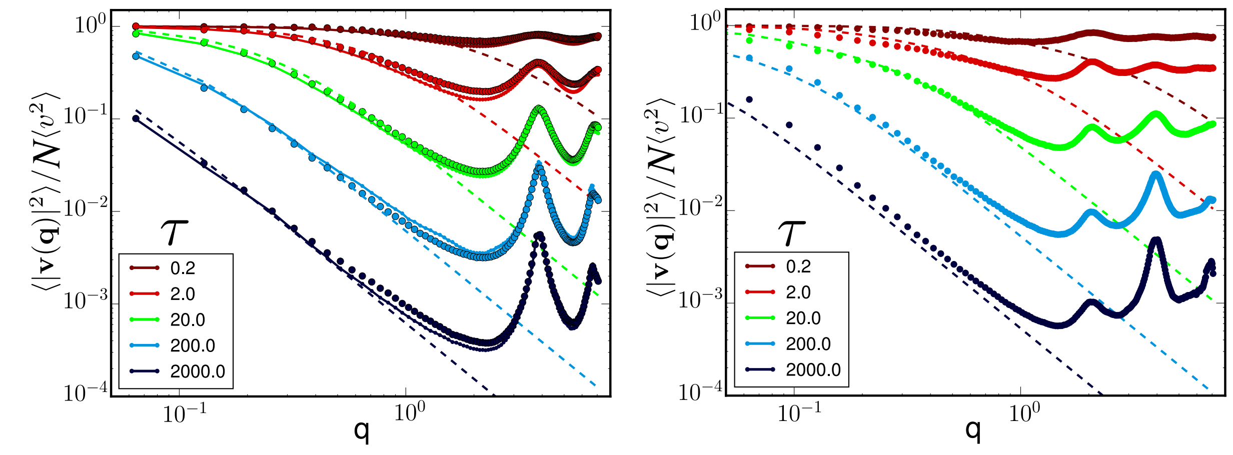

Supplementary Figure 3: Velocity correlations as a function of τ at higher effective temperature $T_{\text{eff}} = 0.02$. Even though most of these systems are slow liquids, the match between simulations, normal modes and elastic predictions remains excellent. Left: Soft disk system. Right: Vertex model

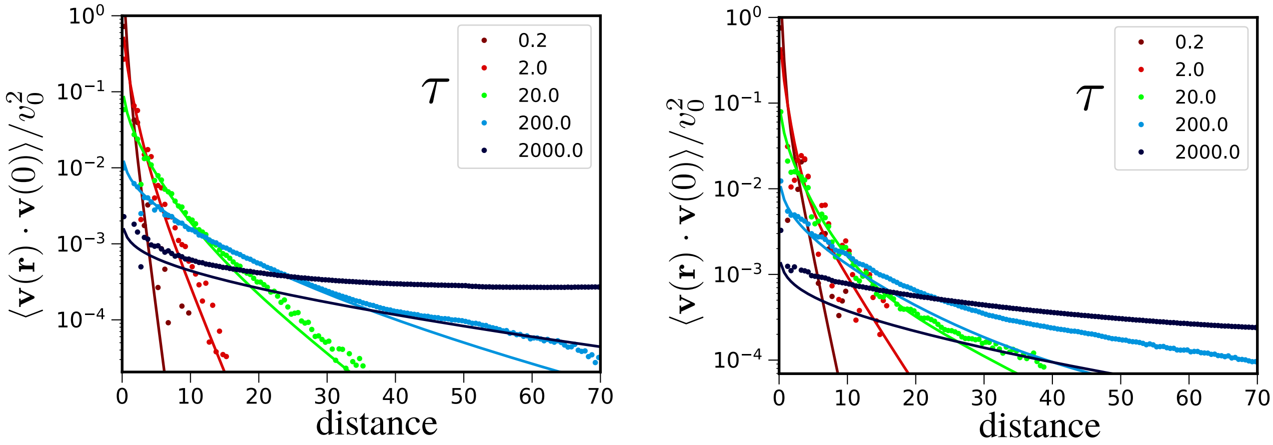

Supplementary Figure 4: Numerical real-space velocity correlations (dots) as a function of τ at effective temperature $T_{\text{eff}} = 0.005$, the counterpart of Fig. 3A in the main text. Lines: Predictions of the analytical result Eq. (14) of the main text. Left: Soft disk system. Right: Vertex model

Supplementary References

[1] P. M. Chaikin and T. C. Lubensky, *Principles of condensed matter physics*, Cambridge University Press (Cambridge, 1995).

[2] L. D. Landau and E. M. Lifshitz, *Mechanics* (Third Edition), Elsevier (1976).

[3] C. E. Maloney, Phys. Rev. Lett. **97**, 035503 (2006).

[4] I.S. Gradshteyn and I.M. Ryzhik, *Table of Integrals, Series and Products*, Fifth edition (Academic Press, 1994).
