# Supplementary material for: Dense active matter model of motion patterns in confluent cell monolayers
Source: Nat Commun. 2020 Mar 16;11:1405. doi: 10.1038/s41467-020-15164-5 (PMC7075903; doi:10.1038/s41467-020-15164-5)
Supplement: Supplementary file 12 — escription of Additional Supplementary [file 41467_2020_15164_MOESM12_ESM.pdf]

Title: Supplementary Movie 1:
Particle model simulation at $T_{eff} = 0.05$ and correlation time $\tau = 200$. Arrows are velocity vectors, colored by velocity magnitude. This simulation is a slow liquid. Other parameters are $\phi = 1$,$k = 1$,$\sigma = 1$,$N = 3183$.

Title: Supplementary Movie 2:
Description: Vertex model simulation at $T_{eff} = 0.005$ and correlation time $\tau = 200$. This simulation is a solid. Other parameters are $K = 1$,$\Gamma = 1$,$A_0 = \pi$,$\bar{p}_0 = 3.6$,$N = 3183$.

Title: Supplementary Movie 3:
Description: Particle model simulation at $T_{eff} = 0.05$ and correlation time $\tau = 2$, showing the near-thermal limit. This simulation is a liquid. Other parameters are $\phi = 1$, $k = 1$,$\sigma = 1$,$N = 3183$.

Title: Supplementary Movie 4:
Description: Vertex model simulation at $T_{eff} = 0.1$ and correlation time $\tau = 2000$, showing the limit where the correlation length reaches system size. This simulation is nominally liquid, but there are no local rearrangements. Other parameters are $K = 1$,$\Gamma = 1$,$A_0 = \pi$,$\bar{p_0} = 3.6$,$N = 3183$.

Title: Supplementary Movie 5:
Description: Human corneal epithelial cells imaged using phase contrast, experiment 7 in the main text. The field of view is 867µm × 662µm, and the cells were imaged over 48 hours. White cells have been extruded out of the sheet.

Title: Supplementary Movie 6:
Description: PIV analysis of experiment 7, arrows are proportional to velocity field and are colored by velocity magnitude.

Title: Supplementary Movie 7

Description: Matched soft disk simulation for the experiments, arrows are the velocity fie ld and are colored by velocity magnitude (in units of µm/h). Parameters are $\phi = 0.95, \frac{k}{\zeta} = 55h^{-1}, \tau = 2.5h, v_0 = \frac{90\ \mu m}{h}, N = 1400$.

Title: Supplementary Movie 8

Description: Same as S7, but with particles colored according to velocity magnitud e, to help visualise particle motion.

Title: Supplementary Movie 9

Matched soft disk simulation for the experiments, with added divisions, with particles colored by velocity magnitude. Parameters are $\frac{k}{\zeta} = 55h^{-1}, \tau = 2.5h, v_0 = \frac{90\mu m}{h}$, and additionally death rate $\frac{1}{a} = 48h$, relative death / division rate $\frac{a}{d} = 0.05$, see ref. [56] for details.

Title: Supplementary Movie 10

Matched vertex model simulation for the experiments. Cells are colored according to their area (in μm^2). Parameters are $A_0 = 380\mu m^2, \frac{\Gamma}{\zeta} = 55h^{-1}, \frac{KA_0}{\zeta} = 55\ h^{-1},\ \tau = 2.5h, v_0 = \frac{90\mu m}{h}, N = 1400$.
