# Supplementary material for: Dense active matter model of motion patterns in confluent cell monolayers
Source: Nat Commun. 2020 Mar 16;11:1405. doi: 10.1038/s41467-020-15164-5 (PMC7075903; doi:10.1038/s41467-020-15164-5)
Supplement: Supplementary file 14 — Source Data [file 41467_2020_15164_MOESM14_ESM.zip › 204078_2_supp_4355968_q472tm.pdf]

Universal velocity correlations in epithelial cell sheets: Computational methods

All the simulations in the article were carried out with the software Soft Active Matter on Surfaces (SAMoS): https://github.com/sknepneklab/SAMoS

Please see the SAMoS code github page for the detailed set of instruction on how to download, compile and install the main simulation code and its analysis toolkit. Please refer to the article Barton, et al. (ref. 38 in the paper) for a detailed description of the self-propelled vertex model (active Voronoi model).

The simulations reported in the article are:

1. Soft disk simulations over an effective temperature, persistence time phase diagram;
2. Vertex model (active Voronoi model) simulation over the same;
3. Vertex and soft disk simulations to match experimental measurements;

This summary will first discuss the first two types of simulations, and their analysis. All scripts used in this process are provided with this summary.

I Simulations

1. Soft disk parameter sweep

Each soft particle simulation is carried out by:

1. Using a custom python script, **random_plane_poly.py**, to create a random configuration of $N = 3183$ particles of mean radius $r = 1$ and 30% polydispersity at packing fraction $\phi = 1$ in a box of area $L \times L = 100 \times 100$, where distances are measured in units of particle radius.
2. The SAMoS configuration file, **makelarge.conf**, which specifies the initial configuration, and sets the simulation parameters, e.g. active driving velocity and noise levels.
3. Executing the configuration file with SAMoS produces output both as plain text files (used for the analysis below) and VTP files, which can be used for visualisation with ParaView[1] [1,2].

We use a BASH shell script to carry these steps out automatically, and save the data in an appropriate folder structure.. The sample script **simulation_disks.sh** will execute a single pair of $T_{eff} = 0.01$, $\nu = 0.01$ for a short test run of 200,000 time steps (full runs were 5 million time steps with the time step $dt = 0.01$).

2. Vertex model parameter sweep

The steps are the same as for the soft disk simulation, with the additional steps that need to be performed before the actual simulation can start:

[1] All simulation images in the paper were created with ParaView.

a) Using a custom python script, **plane_circle_epithelial.py**, to create an initial circular soft particle configuration, with a dense, glued down boundary. The boundary particle information is directly imported into the final vertex simulation.
b) Using SAMoS with the parameter file **equilibration_vertex.conf** to run a short, overdamped soft disk simulation with a glued boundary as an equilibration step. The last positions of the particles act as input for the vertex model simulation.

We provide the shell script **simulation_vertex.sh** to automatically carry out all necessary steps. As for the soft disks, it will create a short test run of 200,000 time steps.

II Analysis

At the core of the analysis are the python analysis functions provided by SAMoS (written by author SH). The analysis structure relevant to the results presented here can be represented by the following diagrams:

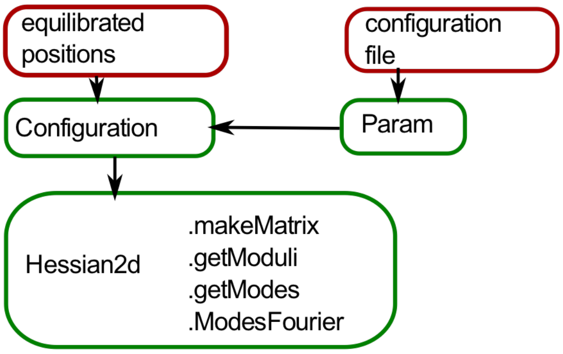

Fig. 1: Normal modes computations

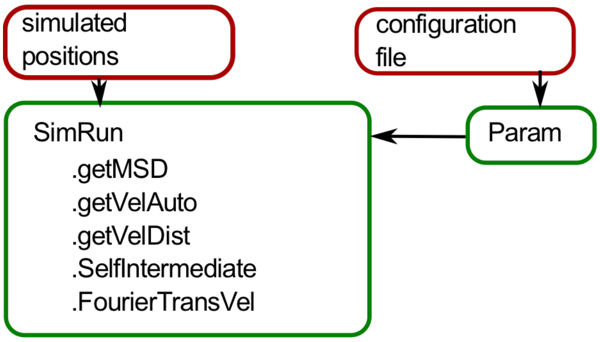

Fig. 2: Correlation computations

1. Normal modes analysis

The expansion into normal modes that we can explicitly perform for the soft disk simulation is as follows:

a) Use the midpoint of the simulation (after 2.5 million steps have been completed) as a starting point to run a steepest descent minimisation of the energy, i.e., a short 100,000 time steps run with no active driving and no thermal noise. This procedure can be done with the sample script **equilibrate_disks.sh**, using **equilibration.conf**, on the data generated above (note that the last step of the simulation serves as starting point for the demonstration script).

b) The positions thus generated, which are in mechanical equilibrium to numerical precision, are the reference positions which we use to numerically construct the Hessian matrix and diagonalise it. This allows us to extract the normal modes and project them in Fourier space. The elastic moduli can also directly be obtained from the Hessian. These functions are implemented in the *Hessian2d* class (see Fig. 1) that is a part of the provided analysis toolkit.

The Hessian is computed by the member function *makeMatrix* of the *Hessian2d* class. As outlined in the Methods section, we can use the Hessian transformed into Fourier space to

compute the elastic moduli of the system. Member function *getModuli* of the *Hessian2d* class returns the longitudinal and transverse projections of the Hessian in Fourier space.
The member function *getModes* of the same class diagonalises the Hessian, and makes its eigenvalues and eigenvectors available for further analysis.

Member function *ModesFourier* of the *Hessian2d* class computes the projection of the individual modes in Fourier space.

The provided script **full_analysis_soft.py** then uses these projected modes to compute the Fourier space velocity correlation function using eq. 6 of the paper.

Note: Since particle positions are off-lattice, this projection cannot be computed using a fast Fourier Tranform, and it is therefore slow. A full projection over all >6,000 modes takes several hours on a high-end workstation (the demonstration script only takes every 100th mode to speed things up – results for the modes are therefore incorrect).

2. Direct simulation analysis

These results can then compared to the actual observed velocity correlation functions. The class *SimRun* (in **Glassy.py**) , see Fig. 2, will read in the data of a full simulation run (with a variable skip at the beginning, usually set at 250 saved snapshots). Its member function *FourierTransVel* will perform the Fourier transform of a single configuration snapshot, and then take its mod square, i.e. the quantity that we can sum to compute the statistically averaged Fourier velocity correlation function.

The script **full_analysis_soft.py** continues the analysis by computing the time averaged Fourier velocity correlation.

SimRun class also contains a number of other member functions that are used in the data analysis in the article:

- *getMSFD* computes the mean square displacement, used to determine if a system is glassy.
- *SelfIntermediate* computes the self-intermediate scattering function. The alpha-relaxation time, when it drops below 0.5, is used as a measure of dynamic slowing down in Fig. 2 of the paper.
- *getVelDist* and *getVelAuto* compute the velocity magnitude distributions, and the velocity autocorrelation functions, respectively. They are used in the comparison between experimental and simulated cell sheets.

The script **full_analysis_soft.py** sequentially computes the elastic moduli, the velocity correlations from normal modes, the mean square displacement, the self-intermediate scattering function, and finally the Fourier space velocity correlations from simulation as well as the analytical result.

For the Vertex model (active Voronoi), we did not compute the normal modes. The script **full_analysis_vertex.py** therefore only computes the mean square displacement, the self-intermediate scattering function, and then the Fourier space velocity correlations from simulation, as well as the analytical result using moduli extracted from Sussman et al., ref 54 in the paper.

The three/two correlation functions are then shown together on a plot, for demonstration purposes.
For this article, we collected the results for each effective active temperature and noise value in a separate python pickle (binary) file. The final plots were then done using a custom script with the collected data.

III Experiment

From the experiment, as described in the methods section, we extract PIV data of the collective cell motion at 10 minute intervals over on average 48 hours. With this summary, we provide the experimental PIV data set corresponding to experiment 7 (shown in supplementary movies 5 and 6 of the article).

The script **PIV_analysis.py** performs the analysis of this data, plots it, and saves it in a pickle file (used for the final plotting).

After defining the field of view of the experiment in microns, in pixels, and in PIV arrows, it reads in the full PIV data set and transforms it into units of microns and hours.
Then, sequentially, it computes and plots:

- The velocities normalised by their instantaneous spatial mean (this improves the robustness of the analysis, as explained in the manuscript), and from there the normalised velocity distributions.
- The temporal velocity autocorrelation function, used to extract the correlation time τ.
- The Fourier transform of the velocities, using the same algorithm as for the simulation data (directly adapted from the member function *FourierTransVel* of the *SimRun* class)
- An approximation of the self-intermediate scattering function. This works by following individual cell patches represented by an arrow backwards in time, a reasonable approximation for small amounts of cell motion.

IV Matching experiment and simulations

The files ending in **expsim** are the scripts and configuration files to run the matching simulations to the experiments. The principle is identical to the soft disk and vertex simulations discussed in section I.

- **run_sheet_nodiv_expsim.sh** performs soft disk simulations matched to the experiment with no cell divisions. It uses the adapted python script **random_plane_poly_expsim.py** to create an initial configuration on the appropriate rectangular experimental field of view with particle areas that match the mean cell area. The SAMoS configuration file **epithelial_nodiv_expsim.conf** is then adapted by the script with the final values for the cell velocity, cell stiffness and correlation time.
- **run_sheet_dividing_expsim.sh** runs matched soft disk simulations with cell divisions. In addition to the parameters of the non-dividing system, the cell division rate is also set by the death rate (in inverse hours) and the attempted division rate (density-dependent). For full details of the dividing model please see our previous publication Matoz-Fernandez, et al. (ref. 56 in the paper). The configuration file for the dividing system is **epithelial_divide_tracers_expsim.conf**.

- **run_sheet_vertex_expsim.sh** runs the matched vertex model simulation of the experiment, with no cell division. Here, we take the mean cell area of the experiment to be the cell target area, and initialise a system of $N = 1{,}500$ particles in a round shape with packing fraction $\phi = 1$, using the adapted python script **plane_circle_epithelial_expsim.py**. As in section I, we equilibrate the soft disk system, here using **equilibration_vertex_expsim.conf**. The parameters for the vertex model in **sheet_vertex_expsim.conf** were found by using dimensional analysis and the stiffness of the soft disk model, as well as the same shape parameter 3.6 as the simulations of section I.

To actually carry out the comparison to experiment, we compute the same quantities for the simulated runs as for the experiment. The python scripts:

- **Collect_correlations_nodiv_expsim.py**
- **Collect_correlations_divide_expsim.py**
- **Collect_correlations_nodiv_vertex_expsim.py**

compute sequentially the simulated Fourier space correlation functions, the temporal velocity autocorrelation function, the self-intermediate scattering function, and finally the velocity distributions. They are then saved in a pickle file for the final comparison. These scripts are based on the *SimRun* class, see section II.2.

Finally, the python script **Plot_correlations_expsim.py** plots all of our results, resulting in the equivalents of Figs. 4A and 4B in the article.

V References

[1] Ahrens, James, Geveci, Berk, Law, Charles, *ParaView: An End-User Tool for Large Data Visualization*, Visualization Handbook, Elsevier, 2005, ISBN-13: 978-0123875822.
[2] Ayachit, Utkarsh, *The ParaView Guide: A Parallel Visualization Application*, Kitware, 2015, ISBN 978-1930934306.
