# Supplementary material for: Dense active matter model of motion patterns in confluent cell monolayers
Source: Nat Commun. 2020 Mar 16;11:1405. doi: 10.1038/s41467-020-15164-5 (PMC7075903; doi:10.1038/s41467-020-15164-5)
Supplement: Supplementary file 14 — Source Data [file 41467_2020_15164_MOESM14_ESM.zip › 204078_2_supp_4350085_q42ffy.gz!/SAMoS/doc/tutorial/tutorial.html]

SAMoS Tutorial


SAMoS Tutorial

- Home- Tissues

- Overview
- Getting Started
  - Download
  - Compile and run
- Simulation setup
  - Input file
  - Configuration file
  - Full Example
- Organisation of the code
- Citing
- Credits
  - Publication


# Overview

SAMoS (Soft Active Matter on Surfaces) is a set of tools for performing numerical simulations of particle-based models for active matter systems.
The package consists of the main simulation program ("samos") and a range of tools for building initial configurations and analysing
results of simulations.

SAMoS has been designed to be modular, simple yet powerful. The code is optimised for speed, but not at the expense of clarity.
The user does not need to be familiar with programming in order to run simulations in SAMoS.

Key features

- Modular design allowing simple setup of complex simulations.
- Simple scripting language for simulation control (no need to modify source code).
- Detailed logging for simple debugging.
- Support for many standard output formats (VTP, mol2, xyz, etc.) for simple visualisation of the results.
- Multiple particle types and particle groups.
- Type/group specific parameters for simulations of multicomponent systems.
- Support for multiple integrators (NVE, Langevin, Browninan etc.) of equations of motion.
- Support for multiple interaction potentials (Lennard-Jones, WCA, soft, Morse, etc.).
- Support for multiple particle alignments (nematic, polar, velocity, Vicsek, etc.).
- Support for simulating filaments (actomyosin) and rods (including confinement of curved surfaces).
- Simulations of systems confined to move on various curved surfaces.
- Simulations of multicomponent epithelial cell layers using Self-propelled Voroni model (SPV).
- Population control (growth, division, death, etc.).

Why yet another particle based code? Unlike many other molecular and Brownian dynamics codes, from the outset SAMoS has been
designed to simulate active matter systems. Therefore, features such as interparticle alignment essential for many
active matter models are included in the core of the code.

#### Examples of systems simulated with SAMoS

|  |  |
| --- | --- |
|  |  |
| Snapshot of the simulation of active polar particles confined to move on the surface of a sphere. | Snapshot of the simulation of a confluent layer of epithelial cells. |

Currently, SAMoS does not support several features that could be of interest for more general and larger simulations. Some of those are
planned for later releases.

**WARNING!** Not supported features

- Hydrodynamic interactions.
- Multi-threaded or MPI parallel execution.
- GPU support.
- Merging of two or more disjointed tissue patches.
- Tissue simulations on curved surfaces.

# Getting Started

In order to use SAMoS, we first need to download the source code and compile it. Currently, only Linux and Mac OS X operating systems are supported.
In principle, it should be possible to compile and run SAMoS on a Windows machine. However, this has not been tested and is not officially supported.

### Downloading SAMoS

Source code of SAMoS package can be downloaded from the GitHub repository.

Clone SAMoS source from GitHub repository

git clone https://github.com/sknepneklab/SAMoS.git

Note: Git is an open-source version control package available on most platforms.

### Compiling and Running SAMoS

#### Requirements

SAMoS is written in C++ using the C++98 standard. Initialisation and analysis tools are written in Python 2.7. The code depends
on several open-source libraries, that are available on most Linux distributions and on Mac via MacPorts.

Required software/libraries

- A modern C++ compiler, such as g++ 4.4 or newer.
- Boost libraries, v. 1.48 or newer.
- GNU Scientific Library (GSL) - version 1.13 or newer.
- CMake (2.8 or newer) - also please install CMake's GUI (ccmake).
- Doxygen - optional but recommended (LaTeX support for building the PDF reference manual).
- VTK library (version 5 or 6).
- CGAL library (version 4.3 or newer). NOTE: Code will fail to compile with CGAL 4.2 or older.
- NumPy, SciPy and maploptlib.

#### Configuring and compiling the code

In order to compile the code, you first need to make sure that all packages and libraries listed above have been
installed on your system.

Configuring and compiling the code (from a console terminal)

1. **cd** *samos* (or the name of the directory you chose to download the code into)
2. **mkdir** *build*
3. **cd** *build*
4. **ccmake** *../*
5. Use the CMake's GUI to select appropriate settings
6. Press '**c**' key several times to configure
7. If all libraries have been found, CMake will allow you to create a Makefile. If not, please exit CMake GUI and
   install missing libraries.
8. Press '**g**' to create Make files. This will terminate CMake's GUI is return you to the shell
9. Type '**make -j 8**' (-j option tells make how many parallel threads to use to compile;
   on a 4-core machine one can typically use 8 threads).
10. If the compilation is successful, an executable '***samos***' should appear in the build directory.
    NOTE: SAMoS uses several libraries in Boost some of which rely heavily on C++ templates. Therefore, compilation may take
    several minutes even on a very fast CPU.

**WARNING!**

Some Linux distributions with newer C++ compilers may have problems with running 8 parallel threads and can
cause the machine to crash. If this happens, use '-j 2', which is likely not to cause any problems on most modern computers.

#### Running simulations with SAMoS

Once the code has been successfully compiled you can use it to run simulations.

Configuring and compiling the code (from a console terminal)

**/path/to/samos/build/samos** *configuration\_file.conf*

*configuration\_file.conf* is a file containing simulations parameters and protocol. It content will be
explained in details in the following sections.

# Simulation setup

In order to run a simulation with SAMoS we need to supply at least two files: a **configuration file** and **input file**.
For filaments simulations, we need to supply one or two additional files, containing connectivity information for bonds and
**angles**.

## Input file

Input file contains information about initial positions, types, sizes, etc. of all particles in the system. It is a text file
that stores required data for each particle as a separate row. Data for each particle is given in columns, separated
by one or more white spaces. Order in which the per-particle data appears is set at the top of the file using **keys:** keyword
followed by a list of keys.

Empty lines or lines stating with the **#** character are ignored.

Example of in input file for 10 particles in the plane

```
# Example of an input file for SAMoS simulation
keys:  id  x  y   nx   ny   vx   vy 
0  -49.812523  -13.220622  -0.557911  -0.829901  -0.064187  -0.997938
1    2.837991  -15.389861  -0.772394  -0.635144   0.841073  -0.540922
2  -28.937555  -17.031011   0.656690   0.754160  -0.871029  -0.491232
3  -43.149352  -17.145390   0.094470  -0.995528  -0.877083  -0.480338
4   -4.272546  -31.464737   0.577069   0.816696   0.204656   0.978834
5  -41.197388  -12.608635  -0.402312   0.915503  -0.785632  -0.618694
6    7.005807   28.411867   0.997299  -0.073445  -0.069671  -0.997570
7   23.550813  -38.901239  -0.124280  -0.992247   0.179116  -0.983828
8   28.584616   -5.643690   0.587347   0.809335  -0.606693  -0.794936
9   44.286823  -48.149627   0.987257   0.159133  -0.959705  -0.281008
```

Keys can appear in any order. However, once set given quantity always must appear in the given column. In other words,
if we set **x** coordinate as the second column, column 2 will be interpreted as **x** coordinate in each row.
Keys that have been omitted will be set to a default value.

List of all available keys

- **id** - unique particle id. Ids are integers starting at 0 (default: 0). Note: This key should always be set.
- **type** - Type of the particle. Type is an integers starting at 1 (default: 1).
- **radius** - particle radius. Radius is a floating point number (default: 1.0).
- **molecule** - molecule this particle belongs to (default: particle id). Note: Used in filament simulations.
- **radius** - particle mass. Mass is a floating point number (default: 1.0).
- **x** - x coordinate of the particle (default: 0.0). Note: User needs to ensure that all particles are inside the simulation box.
- **y** - y coordinate of the particle (default: 0.0). Note: User needs to ensure that all particles are inside the simulation box.
- **z** - z coordinate of the particle (default: 0.0). Note: User needs to ensure that all particles are inside the simulation box.
- **nx** - x component of the director vector (default: 0.0). Note: At least one of the components of the director vector has to be non-zero.
- **ny** - y component of the director vector (default: 0.0).
- **nz** - z component of the director vector (default: 0.0).
- **vx** - x component of the initial velocity (default: 0.0).
- **vy** - y component of the initial velocity (default: 0.0).
- **vz** - z component of the initial velocity (default: 0.0).
- **length** - rod length (for rod simulations) (default: 1.0).
- **ix** - image flag in x direction for unwrapping coordinates (default: 0).
- **iy** - image flag in y direction for unwrapping coordinates (default: 0).
- **iz** - image flag in z direction for unwrapping coordinates (default: 0).
- **nvx** - x component of the vector normal to constraint (default: depends on the constraint).
- **nvy** - y component of the vector normal to constraint (default: depends on the constraint).
- **nvz** - z component of the vector normal to constraint (default: depends on the constraint).
- **area** - native cell area used in tissue simulations (default: 0.0).
- **in\_tissue** - non-zero is particle belongs to a tissue, 0 otherwise (default: 0).
- **boundary** - non-zero is the particle is a tissue boundary particle; 0 otherwise (default: 0.0).
- **parent** - id of the parent used in lineage tracking.

### Bond file

For simulations of flexible and semi-flexible filaments, we also need to supply a file with bond (connectivity information). Bond file is a text file
containing 4 columns. Empty lines and lines starting with **#** character are ignored.

- *column 1* - bond id; ids start from 0.
- *column 2* - bond type; bonds types are integers starting from 1.
- *column 3* - id of the first particle in the bond.
- *column 4* - id of the second particle in the bond.

Note that each bond needs to appear only once.

Example of a bond file with 5 bonds

```
# Example of a bond file
# columns are bond id, bond type, id of 1st particle, id of 2nd particle
0  1  0  1
1  1  1  2
2  1  2  3
3  1  3  4
4  1  4  5
```

### Angle file

For simulations of semi-flexible filaments, we also need to supply a file with angle (bending information). Angle file is a text file
containing 5 columns. Empty lines and lines starting with **#** character are ignored.

- *column 1* - angle id; ids start from 0.
- *column 2* - angle type; angle types are integers starting from 1.
- *column 3* - id of the first particle in the angle (side particle).
- *column 4* - id of the second particle in the angle (angle at this particle).
- *column 5* - id of the third particle in the angle (side particle).

Note that each angle needs to appear only once.

Example of an angle file with 4 angles

```
# Example of an angle file
# columns are angle id, angle type, id of 1st particle, id of 2nd particle, id of 3rd particle
0  1  0  1  2
1  1  1  2  3
2  1  2  3  4
3  1  3  4  5
```

### Tissue boundary file

Tissue simulations require a file which sets connectivity of the boundary particles. This is a text file
containing 3 columns. Empty lines and lines starting with **#** character are ignored.

- *column 1* - boundary bonds id; ids start from 0.
- *column 2* - id of the first particle in a tissue boundary bond.
- *column 3* - id of the second particle in a tissue boundary bond.

Note that each bond needs to appear only once.

Example of a boundary connectivity file

```
# Example of a boundary connectivity file for tissue simulations
# columns are bond id, id of 1st particle, id of 2nd particle
0  11 17
1  17 25
2  25 32
3  32 11
```

## Configuration file

Configuration (or parameter) file (script) is a text file which sets the parameters (such as interaction types, integrators, etc.) as well as
the simulation protocol. Commands in the file are executed in the order in which appear. Commands are executed in "interpreter"-like fashion,
meaning that the configuration file is parsed one line at the time. Each line is executed right away and the program does not
continue to the next command until the current command has completed its execution.

SAMoS uses a simple parser to parse commands in the configuration script. Commands cannot be split over multiple lines and there can be only
one command per line.

General syntax for commands is:

```
    command_name [arg] { param_1 = val_1; param_2; param_3 = val_2 }
```

where *command\_name* is the command, *arg* is one or zero arguments, depending on the command, followed by a list of semi-colon separated
parameters. Parameters can appear either as single keywords or as argument-value pairs. As in the case of input file, empty lines and lines starting with
the **#** characters (comments) will be ignored.

Example of a configuration file

```
# All runtime messages will be sent to file messages.msg
messages messages.msg

# Every simulation requires a box
# "periodic" means that periodic boundary are applied.
# box is cubic with size 100x100x10 (units of particle radius r = 1) and spans from -50 to 50 in
# x and y directions and -5 to 5 in z direction
box periodic { lx = 100.0;  ly = 100.0;  lz = 10.0 }

# We read in particle positions from file plane.dat (generated by tools in utils directory)
input plane.dat

# We set the neighbour list with cutoff distance 2.4 and padding distance of 0.5
nlist { rcut = 2.5; pad = 0.5 }

# Output particle velocities. Base name of the file is plane_test
# File extension will we '.vtp'. Output will be generated every 10000 time steps
# staring from the step 0. Each snapshots will be stored in a separate file (keyword 'multi')
# with name plane_[time_step].vtp (e.g., plane_0000010000.vtp)
# and each file with contain a header with the description of its content
dump plane { type=vtp; start=0; freq=10000; multi }

# We constrain all particles to move only on the xy plane
constraint plane {  }

# All particles are interacting with the soft-core potential of strength
# k = 1.0 (energy units)
# a = 2.0 (distance units)
pair_potential soft { k = 1.0; a = 2.0 }

# Alignment will be done using polar aligner
# Coupling constant J = 1.0 (energy units)
pair_align polar { J = 1.0 }

# We would also like to monitor measure a few quantities
# Here we define a log that keeps track of step, velocity, soft core potential energy, and alignment
# These quantities will be logged once every 1000 steps
# Note that step is always logged automatically
log plane.log { freq = 1000; velocity; soft_energy; polar_align }

# we set the simulation time step to 0.01
timestep 0.01

# In order to relax the system and remove large overlaps
# we first run system using NVE integrator with maximum
# displacement limit=0.001. 
integrator nve { limit=0.0001 }

# Run for 10,000 steps
run 10000

# Relaxation is done. We need set disable the NVE integrator
disable nve { group=all }

# Now we are at the point to execute actual simulations.
# We switch the integrator to brownian.
# Integrate equations of motion using Brownian dynamics.
# seed is the initial seed for the random number generator
# nu sets the width of the distribution for random changes of velocity
# mu is particle mobility
# v0 is the intensity of the self-propelling velocity
# we apply integrator to group "all" (i.e, to all particles in the system)
integrator brownian { seed = 1;  nu = 0.02; mu = 1.0;  v0 = 0.05; group = all }


# Finally, we run simulation for 100,000 time steps
run 100000
```

## Step by Step guide for setting up a simple simulation

In this section we will go over necessary steps to set up a simple simulations to study $N$ monodisperse polar active
particles on the surface of a sphere of radius $R=30$ with packing fraction $\phi=0.7$.

### Step 1: Building initial configuration

We start by building the initial configuration for our simulation. This can be done using the **random\_sphere.py** Python script
provided in the *samos/configurations/MakeConfigurations* directory.

Make initial configuration for simulating particles on a sphere

```
python /path/to/samos/configurations/MakeConfigurations/random_sphere.py -R 10.0 -f 0.7 -o sphere.dat
```

This will create an initial configuration file (input file) called sphere.dat that will
contain ids, types, radii, position, initial velocities and inital velues of the director field for $N=316$ particles. All particles will have
same radius ($r=0.5$) and same type (1). Director field will be point in a random direction, but it will be tangential to the sphere at every point.
Velocity vectors will also point along the random direction tangent to the sphere and will have magnitude $|\vec v|=1$.

Now that we have initial configuration, we can move on an create a configuration file to run this simulation. We will call this file *soft\_on\_sphere.conf*.

### Step 2: Adding message and metadata files (optional)

In order to create a file with information, warning and error message produced by the simulation we use the command

Adding messanger

```
message messages.msg
```

All messages regarding the state of the simulation will we sent to the file *messages.msg*. This file will be in the same working directory that
the simulation uses to output all other results.

SAMoS also supports automatic generation of the meta-data for curating the results of simulations. These can be stored in several formats including JSON and XML.
Meta-data is produced by the *config* command. Note that the meta-data file will be produced only upon successful termination of the code. In other words,
if the code fails, or if it is interrupted, no meta-data file will be generated.

Adding metadata (JSON format)

```
config sphere { type = json }
```

Previous command will generate a file named *sphere.json* in the simulation execution directory.

### Step 3: Setting the simulation box

In order to run a simulation, we need to specify the simulation box and the boundary conditions (fixed or periodic). SAMoS assumes that the
simulation box is centred at the origin. That is, if the box has dimensions $\left(L\_x,L\_y,L\_z\right)$ allowed particle coordinates will be
$-L\_x/2\le x < L\_x/2$, $-L\_y/2\le y < L\_y/2$, $-L\_z/2\le z < L\_z/2$.

Setting a simulation box with fixed boundary conditions

```
box fixed { lx = 30.0; ly = 30.0; lz = 30.0 }
```

Note that the user needs to ensure that the all particles coordinates in the input file are within the simulation box. If this is not the
case, SAMoS will return an error and terminate.

### Step 4: Reading input data

In this step we need to supply the input file (initial configuration) to SAMoS.

Reading input file

```
input sphere.dat
```

Previous command loads input configuration generated in Step 1 and prepares it for simulation.

### Step 5: Setting the neighbour list

In order to speed up calculation of short-range forces, SAMoS uses standard cell and neighbour list algorithms. It is therefore necessary
to specify parameters such as neighbour list cutoff and padding distance.

Setting the neighbour list parameters

```
nlist { rcut = 2.4; pad = 0.5 }
```

Previous command sets the cutoff distance to $r\_c=2.4$ (recall that the potential range is $2r = 1.0$) and padding distance ($r\_p=0.5$). Neighbour list
will be rebuilt once one of the particles travelled more that $r\_p/2$.

### Step 6: Output simulation results

In this step we supply so-called *dumps*. That is, we specify format and frequency of outputting simulation results. We can specify as many dumps
as necessary. However, keep in mind that some of dump types can be slow (e.g., VTP) and rather large, which may result is generating a huge amount of data
is hinder simulation performance.

Here we specify two dumps, one into VTP files and other one into a regular, text-based file that can be directly used as an input for a next run. In both cases,
dumps are split over multiple files (labelled by the time step) and produced every 1000 time steps.

Setting the neighbour list parameters

```
dump sphere_example { type = vtp; start = 0; freq=1000; multi }
dump sphere_example { type=full; start=0; freq=1000; multi; id; type; radius; coordinate; velocity; director; keys  }
```

First command will produce a sequences of VTP files with names sphere\_example\_0000XX000.vtp (ready to be visualised with ParaView). Second command
will produce a sequence of plain text files with names sphere\_example\_0000XX000.dat, with the **keys:** keyword and the list of column names
at the top.

### Step 7: Specifying the constraint

In this example we study active particles confined to move on the surface of the sphere. Therefore, we need to tell SAMoS to use the
spherical constraint.

Specifying spherical constraint

```
constraint sphere { r = 10 }
```

### Step 8: Setting interparticle interactions

In this example, we'll assume that particles interact with each other via a short-range soft repulsive potential of the form
$$V(r) = \frac{k}{2}(r-a)^2$$ for $r < a$ and $V(r)=0$ otherwise. In the last equation, $r$ is the Euclidean distance between particles
$i$ and $j$, i.e., $r=\left|\vec r\_i - \vec r\_j\right|$, $k$ is the elastic constant and $a$ is the potential range.

Setting the interaction potential between particles

```
pair_potential soft { k = 10.0; a = 1.0 }
```

### Step 9: Setting interparticle alignment

In many active particle simulations it is not only sufficient to know the particle position, but one need to know the direction of the internal
degree of freedom, i.e. the director. Alignment interactions calculate torques on the internal degree of freedom. In this example, we assume that
particles are polar and that the align to their neighbours within cutoff $r\_c$ (set by the neighbour list). Polar alignment assumes simple XY-model
coupling, i.e. it tries to minimise energy functional
$$ E\_{polar} = -J\sum\_j \vec n\_i\cdot\vec n\_j $$
where $\vec n\_j$ are directors of the particles within radius $r\_c$ of particle $i$ and $J$ is the coupling constant.

Setting the alignment between particles

```
pair_align polar { J = 1.0 }
```

### Step 10: Logs (optional)

It is often useful to monitor global properties of the system such as kinetic energy or total potential energy. In order to do so, we define a *log*
which will contain such information. We output log information once every 100 time steps. Logs will be written into the file *sphere\_test.log*.

Logging simulation state

```
log sphere_test.log { freq = 100; velocity; soft_energy; polar_align }
```

### Step 11: Time step

In order to be able to integrate equations of motion, we need to set the simulation time step. SAMoS supports multiple integrtors that simultaneously
can act on different groups or degrees of freedom (e.g., one integrator updating particle positions and the other updating the orientation of the director).
Therefore, in order to be able to ensure consistent simulation results, we need to specify a global simulation time step.

Setting global time step

```
timestep 0.01
```

### Step 12: Relaxation (optional)

Python script we used in Step 1 to generate the initial configuration did not check if there are large overlaps between particles. If this is the case, which
is almost always true for dense systems, running the simulation starting from such configuration would result in very large forces that can lead to a crash.
This is particularly the case is one works with hard-core repulsive forces. In the case of soft potentials, as in this example, the effects of overlaps are
less severe. However, it is a good practice to "relax" the system before starting the actual run. We achieve this by using the *NVE* integrator which
limits the maximum distance a particle can move, regardless of the force acting on it.

NVE with limited step

```
integrator nve { limit = 0.0001  }
```

### Step 13: Relaxation (continued)

We proceed to run the simulation for 10,000 time steps, in order to remove large inter-particle overlaps.

Relaxation run

```
run 10000
```

### Step 14: Relaxation (completed)

Now that we have completed the relaxation run, we need to disable NVE integrator with the limited step size, in order to prepare the system for actual
simulation.

Disabling the integrator

```
disable nve { group = all }
```

### Step 15: Self-propulsion

We are finally ready to simulate the actual active system. In order to do so, we need to introduce self-propulsion. Self-propulsion acts on
individual particles and does not depend on the surrounding. Therefore, it is categorised as an external (single-particle) effect. We assign
activity of strength $\alpha = 1.0$ acting on each particle in the direction of its director.

Specifying activity

```
external self_propulsion { alpha = 1.0 }
```

### Step 16: Integrators

In this step we set the integrators for the equations of motion. Since each particle contains the director, which acts as an internal degree of freedom
we need to specify two integrators, one for particle positions and one for the orientation of the director. In both cases we'll use simple, first order
Brownian dynamics integrators. However, in case of the particle position we'll also introduce thermal noise at temperature $T=0.1$. Director will be subject
to a random noise of strength $\nu = 0.01$.

Setting integrators for position and orientation

```
integrator brownian_pos { seed = 0; mu = 1.0;  temperature_control = constant; min_val = 0.1 }
integrator brownian_align { seed = 0; nu = 0.01;  }
```

### Step 17: Running the simulation

We are finally ready to run the simulation. We'll run it for 100,000 time step.

Running the simulation

```
run 100000
```

### Summary

Above example will produce 111 VTP files, 111 dat files, one log file, one message and one JSON file. All files will be stored in the execution directory, which is the
same directory that contains the configuration and input files.

This completes the simulation part. One can now use Paraview (or any other visualisation software) to visualise results of the simulation. In addition,
one can use the data stored in "dat" files in order to extract various physical quantities. Some of the analysis tools are available in the *utils*
directory. Alternatively, the user might want to write her own analysis code.

Follow the links for download complete configuration and input
files used in this example.

# Organisation of the code

The layout of the code structure is shown in the figure below.

# Credits

SAMoS is developed in the School of Science and Engineering and the School of Life Sciences at the University of Dundee, United Kingdom. Scientific projects that use
SAMoS code in research have been funded by EPSRC and BBSRC.

- Rastko Sknepnek (University of Dundee) -  **lead designer and developer**

Code contributions by:

- Silke Henkes (University of Aberdeen) - numerous initial configuration building and data analysis scripts
- Daniel Barton (University of Dundee) - tissues simulation setup and analysis scripts
- Amit Das (National Institute for Biological Sciences) - setup of actomyosin simulations

## Publications

Published papers that use SAMoS:

1. R. Sknepnek and S. Henkes, *Active swarms on a sphere*, Phys. Rev. E **91**, 033205 (2015).

SAMoS (c) 2013-2016, University of Dundee
