# Supplementary material for: Dense active matter model of motion patterns in confluent cell monolayers
Source: Nat Commun. 2020 Mar 16;11:1405. doi: 10.1038/s41467-020-15164-5 (PMC7075903; doi:10.1038/s41467-020-15164-5)
Supplement: Supplementary file 14 — Source Data [file 41467_2020_15164_MOESM14_ESM.zip › 204078_2_supp_4350085_q42ffy.gz!/SAMoS/doc/tutorial/tissues.html]

SAMoS Tutorial


SAMoS Tutorial

- Home
- Tissues

- Overview
  - Active Vertex Model
  - Handling boundaries
  - Boundary forces
  - Equations of motion
  - Cell division and death
  - Comparison with SPV
- Examples
  - Fixed boundary
  - Growth and division
  - Multiple cell types
- References


# Overview

SAMoS implements an extended version of the recently introduced Self-propelled Voronoi model (SPV) by 
Bi, et al.. Current implementation allows for open (fixed and dynamic) boundaries, multiple cell types, as well as cell division and death. Due to
these differences, we refer to the tissue model implemented in SAMoS as *Active Vertex Model* (AVM).

## Active Vertex Model

The main idea behind the AVM model is that one makes a mapping between the Vertex model widely used for describing tissues and the particle based model
for active matter. This allows to perform large-scale simulations of tissues and include a wide variety of local cellular movement in a natural fashion.
The basic unit in our model is a particle, whose position can be thought as approximately corresponding to the centre of mass of the cell.
Tissue is represented as a Voronoi tesselalation of the plane. Therefore, in this approach, epithelial cells are interpreted as Voronoi cells
corresponding to the cell centres, i.e., particle positions.

In the vertex model, energy is defined on vertices and edges. It is therefore necessary to make connection between the cell-centre based description
of the SPV model and the quantities defined on the vertices of the Voronoi diagram.

In the vertex model energy of the tessellation is defined as
\begin{equation}
E\_{vertex}=\sum\_{i=1}^{N\_{face}}\left[\frac{K\_{i}}{2}\left(A\_{i}-A\_{i}^{0}\right)^{2}+\frac{\Gamma\_{i}}{2}P\_{i}^{2}\right]+\sum\_{\mu,\nu}\Lambda\_{\mu\nu}l\_{\mu\nu},
\label{eq:vertex\_model\_energy}
\end{equation}
where the first sum goes over all faces (cells), $K\_{i}$ is the cell stiffness, measuring resistance to change the cell area $A\_{i}$ from its native area $A\_{i}^{0}$.
$\Gamma\_{i}$ measures the resistance to changing the cell perimeter, $P\_{i}$. $l\_{\mu\nu}$ is the length of the edge connecting vertices $\mu$ and $\nu$ (on the Voronoi
mesh) and $\Lambda\_{\mu\nu}$ is the resistance of changing the contact length between two neighbouring cells, i.e., the tension of the cell junctions.

**Fig. 1:** Two coordinate representations. In our model we track particles that correspond to cell centres (yellow spheres).
Particle positions are denoted using latin indices. Particles form a Delaunay triangulation (blue lines). Dual of this triangulations is a
Voronoi diagram, with each Voroni cell representing actual cell. Cell edges are marked with red lines. Vertices of the dual mesh are denoted by Greek indices.

In Figure 1 we show two representations used in this model. We are going to adhere to a convention that Latin indices denote particle positions and
Greek indices label vertices of the Voronoi network. In this description, area corresponding to the particle i is nothing but the area of the polygon
(face) on of the vertex mesh surrounding site i and is given as,
\begin{equation}
A\_{i}=\frac{1}{2}\sum\_{\mu}\left(\vec{r}\_{\mu}\times\vec{r}\_{\mu+1}\right)\cdot\vec{N}\_{i},
\end{equation}
where $\vec{r}\_{\mu}$ is the position of vertex $\mu$ and $\vec{N}\_{i}$ is a unit-length vector perpendicular to the surface the polygon
and it does not depend on the position of vertices. This is expression is just a discrete version of the Green's formula.
We will assume that the triangulation is Delaunay and vectors $\vec{r}\_{\mu}$ correspond to the vertices of the dual Voronoi lattice.

**Fig. 2:** Definition of the circumcenter of a non-obtuse triangle.

For a given Delaunay triangle, the position of the Voronoi centre is given as (see Fig. 2)
\begin{equation}
\vec{r}\_{\mu}=\frac{\lambda\_{1}}{\lambda\_{1}+\lambda\_{2}+\lambda\_{3}}\vec{r}\_{i}+\frac{\lambda\_{2}}{\lambda\_{1}+\lambda\_{2}+\lambda\_{3}}\vec{r}\_{j}+\frac{\lambda\_{3}}{\lambda\_{1}+\lambda\_{2}+\lambda\_{3}}\vec{r}\_{k},
\label{eq:r\_mu\_baricentric}
\end{equation}
where we use the notation
\begin{eqnarray}
\lambda\_{1} &=&l\_{i}^{2}\left(l\_{j}^{2}+k\_{k}^{2}-l\_{i}^{2}\right) \\
\lambda\_{2} &=&l\_{j}^{2}\left(l\_{k}^{2}+l\_{i}^{2}-l\_{j}^{2}\right) \\
\lambda\_{3} &=&l\_{k}^{2}\left(l\_{i}^{2}+l\_{j}^{2}-l\_{k}^{2}\right),
\end{eqnarray}
where
\begin{eqnarray}
l\_{i} &=&\left|\vec{r}\_{j}-\vec{r}\_{k}\right|=\left|\vec{r}\_{jk}\right| \\
l\_{j} &=&\left|\vec{r}\_{k}-\vec{r}\_{i}\right|=\left|\vec{r}\_{ki}\right| \\
l\_{k} &=&\left|\vec{r}\_{i}-\vec{r}\_{j}\right|=\left|\vec{r}\_{ij}\right|.
\end{eqnarray}

In order to implement the AVM into a particle based code such as SAMoS, we have to compute forces acting on each particle (cell-centre in this case).
This is done directly by computing the negative derivative of the energy functional in Eq. (\ref{eq:vertex\_model\_energy}). The only
real complication is that the energy is most naturally written in terms of positions of the Voronoi vertices, while the basic degrees of freedom
in AVM are the cell-centres. Therefore, we need to apply the chain rule in order to extract forces on a particle. In general,
\begin{equation}
\vec{F}\_{i}=-\nabla\_{\vec{r}\_{i}}E\_{vertex}.\label{eq:general\_force-1}
\end{equation}
Another complication we need to worry about is that moving particle $i$ will change area of its neighbouring cells and thus exert force
on a number of other particles in its surrounding. All those contributions have to be taking into account when computing the force. A direct
consequence of this is that the **forces on cell centres cannot be written as simple pairwise interactions**. However, they still remain local
and only nearest neighbours contribute to the total force.

After a lengthy, but straightforward calculation we derive to the final expression for the force on particle (cell) $i$,
\begin{eqnarray}
\vec{F}\_{i} & = & \vec{F}\_{i}^{a}+\vec{F}\_{i}^{p}+\vec{F}\_{i}^{j}\nonumber \\
& = & -\sum\_{k=1}^{N\_{part}}\frac{K\_{k}}{2}\left(A\_{k}-A\_{k}^{0}\right)\sum\_{\nu\in l\_{k}}\left[\vec{r}\_{\nu+1,\nu-1}\times\vec{N}\_{k}\right]^{T}\left[\frac{\partial\vec{r}\_{\nu}}{\partial\vec{r}\_{i}}\right]\label{eq:total\_force}\\
& & -\sum\_{k=1}^{N\_{part}}\Gamma\_{k}P\_{k}\sum\_{\nu\in l\_{k}}\left(\hat{\vec{r}}\_{\nu,\nu-1}-\hat{\vec{r}}\_{\nu+1,\nu}\right)^{T}\left[\frac{\partial\vec{r}\_{\nu}}{\partial\vec{r}\_{i}}\right]\\
& & -\sum\_{k=1}^{N\_{part}}\sum\_{\nu\in l\_{k}}\left[\Lambda\_{\nu-1,\nu}\hat{\vec{r}}\_{\nu,\nu-1}-\Lambda\_{\nu,\nu+1}\hat{\vec{r}}\_{\nu+1,\nu}\right]^{T}\left[\frac{\partial\vec{r}\_{\nu}}{\partial\vec{r}\_{i}}\right].
\label{eq:avm\_force}
\end{eqnarray}

In previous equation $\left[\frac{\partial\vec{r}\_{\nu}}{\partial\vec{r}\_{i}}\right]$ is the $3\times3$ Jacobian matrix for changing coordinates from
the vertices to cell centres.

We note that the $k$ sum runs over all immediate neighbours of cell $i$, hence leading to the expression for the force that is not pair-wise.
In order to see explicitly what goes into computing the force on cell $i$, we focus only of the area, $\vec F^a$, term.

We loop over all cells surrounding cell $i$.

- For particle $i$ compute $\frac{K\_{i}}{2}\left(A\_{i}-A\_{i}^{0}\right)$ and multiply it with the sum
  $\sum\_{\nu\in l\_{i}}\left[\left(\vec{r}\_{\nu+1}-\vec{r}\_{\nu-1}\right)\times\vec{N}\_{i}\right]^{T}\left[\frac{\partial\vec{r}\_{\nu}}{\partial\vec{r}\_{i}}\right]$.
  Note that this sum runs over all vertices (corners) $\nu$ of the cell $i$.
- For all immediate neighbours $j$ of cell $i$ compute $\frac{K\_{j}}{2}\left(A\_{j}-A\_{j}^{0}\right)$ and multiply it with the sum
  $\sum\_{\nu\in l\_{i}\cap l\_{j}}\left[\left(\vec{r}\_{\nu+1}-\vec{r}\_{\nu-1}\right)\times\vec{N}\_{j}\right]^{T}\left[\frac{\partial\vec{r}\_{\nu}}{\partial\vec{r}\_{i}}\right]$.
  Note that $\nu\in l\_{i}\cap l\_{j}$ ensures that vertices $\nu$ surrounding $j$ are taken into account only if the are
  affected by (also belong to) the cell $i$. That is, we only consider those $\vec{r}\_{\nu}$ for which coordinates $\vec{r}\_{i}$ of cell $i$ appear in
  Eq. (\ref{eq:r\_mu\_baricentric}).

Note that quantities such as the native area, $A\_{0}$ are not easily defined for the boundary cells (particles). Therefore, it is clear
that boundary particles have to be treated separately.

## Handling Boundaries

**WARNING!**

Current implementation of the AVM does not support periodic boundary conditions.

The most general method that would allow changes of topology of the boundary, such as parts of the tissue being allowed to detach or holes
being allowed to open and close, is not an easy problem. It would require complex checks and modifications of the underlying Delaunay
triangulation, which would introduce a non-negligible additional computational cost on top of already elaborate mechanisms required to maintain and
dynamically update the triangulation. Therefore, in current implementation we assume that the topology (connectivity) of the boundary is maintained
throughout the entire simulations. This does not mean that the boundary is fixed. It can grow, shrink and change its shape, but it cannot
change its topology, e.g. it is not possible transition from a disk to an annulus. Examples of allowed and disallowed changes of the boundary
are shown in Fig. 3.

**Fig. 3:** Schematic representation of the allowed and disallowed changes of the boundary. Initial configuration with the topology of a disk (a)
is allowed to develop pronounced fingers (b). However, it is not possible to split into two domains (c) or develop a hole (d).

In the AVM the degrees of freedom are particles that represent cell centres. These particles also serve as sites of the Delaunay triangulation.
In order to handle boundaries we introduced two types of particles, *boundary* and *internal*. In addition we specify the connectivity
between boundary particles. Boundary particles together with their connection information form a *boundary line*. This line sets the topology
of the boundary, which is preserved throughout the simulation, and delineates between the tissue and its surrounding. In addition, the boundary line can
have free energy, such as line tension or bending stiffness, assigned to it. Biologically, this would correspond to the complex molecular
For simplicity, we assume that each boundary particle has exactly two boundary neighbours. This prevents somewhat pathological,
"cross-like" configurations where two otherwise disjoint domains would hinge on a single boundary site.

Boundary particles do not represent centres of actual cell. In a Delaunay triangulation, star of the boundary vertex is incomplete, which introduces
a number of complications related to properly defining quantities such as the associated dual area or perimeter. These quantities can
unambiguously be defined only for the internal particles, which in turn correspond to cells. Boundary particles instead can be thought
of as "ghosts" that serve to model the complex processes that occur at the buffer layer between cells in the epithelial sheet and
its environment. These particles, however, experience forces from the interior of the tissue as well as form the interactions with their
neighbours on the boundary (such as line tension and bending rigidity). The boundary line, dynamically adjusts its shape to the cell configuration
inside the tissue.

Due to the dynamic nature of the model, even without cell and death, it is not possible to retain a constant number of boundary
particles. Instead, the boundary line has to be able to contract or extend in order to accommodate changes inside the tissue. This is
achieved by adding and removing boundary particles. Here we outline the algorithm used to achieve this.

We first focus on the boundary expansion. We require that that all cells are contained within the boundary, that is that no cell vertices
spill over to the outside region. The spill-over happens if the angle opposite to a boundary edge is greater than $90^{\circ}$. In this
case, the centre of the circumscribed circle falls outside the triangle and, therefore, outside the boundary. In order to prevent this from happening we
perform following check (see Fig. 4):

1. For each boundary edge $e$ compute angle $\alpha\_{e}$ at the internal particle $p\_{e}$ opposite to it.
2. If $\alpha>90^{\circ}$
   1. Compute position, $\vec{r}\_{p\_{n}}$, of the new particle $p\_{n}$ by mirroring, $\vec{r}\_{p\_{e}}$, coordinates of $p\_{e}$ with
      respect to edge $e$. If $\vec{\hat{r}}\_{e}$ is the unit-length vector along edge $e$ then
      \begin{equation}
      \vec{r}\_{p\_{n}}=2\left(\vec{r}\_{p\_{e}}\cdot\hat{\vec{r}}\_{e}\right)\hat{\vec{r}}\_{e}-\vec{r}\_{p\_{e}}.
      \end{equation}
   2. Add new boundary particle $p\_{n}$ at position $\vec{r}\_{p\_{n}}$ and mark it as boundary.
   3. Remove boundary edge $e$, i.e., two boundary particles at its end are no longer neighbours.
   4. Connect $p\_{n}$ to the two boundary particles disconnected in (c).
   5. Connect $p\_{n}$ to $p\_{e}$.
3. If at least one new boundary particle was added in 2., rebuild the triangulation.

Note that the procedure outlined above always converges in a single step. A boundary edge is flipped is the angle opposite to it is $90^{\circ}$.
The flip creates two new triangles that are both acute at the internal vertex and therefore cannot trigger additional flips in the same step.

**Fig. 4:** Expansion of the boundary by adding a new boundary particle. (a) If the angle at the internal particle shaded in green opposite to the
highlighted edge reaches $90^{\circ}$, one of the corners of the cell (yellow polygon) touches the highlighted edge. This triggers a "flip" mechanism.
(b) The internal particle shaded in green is mirrored along the shaded edge in (a) and a new boundary particle (top blue) is introduced.
The shaded edge is flipped such that the new particle is connected to the "green" one.

Shrinking of the boundary is achieved by removing particles that have no connections to the internal particles, i.e., they only have two
edges connecting them to the two neighbouring boundary particles. In other words, in this situation, no part of a cell can be inside
the triangle having two of its sides being boundary edges and it can be safely removed. The algorithm schematically outlined in Fig. 5 is as follows:

1. For each boundary particle $p$ compute number of edges $n\_{e}\left(p\right)$ that the particle belongs to.
2. If $n\_{e}\left(p\right)\le2$ remove $p$.

Note that the position of the "dangling" particles does not directly affect the shape of the cells and, therefore, if is not essential
for it to be removed right away. Instead, "dangling particles are removed only when during neighbour list rebuilds.

An important point to make is that the algorithms proposed for adding and removing boundary particles **do not** lead to any jumps in
the shape and force of the cells. However, both addition and removal of boundary particles will lead to jumps in the boundary forces, such
as those resulting from line tension or bending. These jumps are inevitable due to the discontinuous nature of the extension and shrinkage of
the boundary. However, in practice, such jumps are relatively small and do not lead to any appreciable artifacts in the cell dynamics.

**Fig. 5:** Shrinking of the boundary is achieved by removing boundary vertices that have only two bonds. (a) If the sum of angles at two particles
shaded in green opposite the edge connecting the internal and boundary particles is greater than $180^{\circ}$ the edge is flipped (this
is the standard equangulation move that occurs for all internal edges). (b) After the flip, the boundary particles shaded in green has only
two bonds, both to its boundary neighbours. (c) The "green" particle in (b) is removed.

## Boundary forces

It is plausible to assume that the boundary of an epithelial cell sheet will experience additional forces due to its resistance to stretching
(line tension) and bending. For simplicity, we model those effects by measuring distances and angles between centres of neighbouring boundary
particles. In case of line tension, we write
\begin{equation}
E\_{lt}=\frac{1}{2}\sum\_{\left\langle i,j\right\rangle }\lambda\_{i,j}\left(l\_{ij}-l\_{0}\right)^{2},
\end{equation}
where $\lambda\_{i,j}$ is the line tension of the edge connecting boundary particles $i$ and $j$, $l\_{ij}=\left|\vec{r}\_{i}-\vec{r}\_{j}\right|$
is the length of that edge and $l\_{0}$ is the length of an non-stretched bond.

We can directly compute force on boundary particle $p$ due to line tension, by evaluating $\vec F^{lt}\_p = -\nabla\_{\vec r\_p}E\_{lt}$, which is
\begin{equation}
F^{lt}\_p = -\lambda\_{p,p+1}\left(\left|\vec{r}\_{p,p+1}\right|-l\_{0}\right)\hat{\vec{r}}\_{p,p+1}-\lambda\_{p-1,p}\left(\left|\vec{r}\_{p,p-1}\right|-l\_{0}\right)\hat{\vec{r}}\_{p,p-1}.
\end{equation}

We model bending of the boundary as
\begin{equation}
E\_{bend}=\frac{1}{2}\sum\_{i}\kappa\_{i}\left(\theta\_{i}-\theta\_{0}\right)^{2},
\end{equation}
where $\kappa\_{i}$ is the stiffness of the angle $\theta\_{i}$ at the boundary particle $i$. This angle is given as
\begin{equation}
\theta\_{i}=\arccos\frac{\vec{r}\_{ji}\cdot\vec{r}\_{ki}}{\left|\vec{r}\_{ji}\right|\left|\vec{r}\_{ki}\right|},
\end{equation}
where $\vec{r}\_{j}$ and $\vec{r}\_{k}$ are positions of boundary vertices to the left and to the right of vertex $\vec{r}\_{i}$.

In order to compute the force one needs to compute negative gradient with respect to the position of boundary particle $p$. After a lengthy but
straightforward calculation, we obtain the force $\vec F^{bend}\_p$ as

\begin{equation}
\vec F^{bend}\_p = \vec{F}\_{p}^{\left(1\right)} + \vec{F}\_{p}^{\left(2\right)} + \vec{F}\_{p}^{\left(3\right)},
\end{equation}
with
\begin{equation}
\vec{F}\_{p}^{\left(1\right)}=-\left(\vec{F}\_{p-1}^{\left(2\right)}+\vec{F}\_{p+1}^{\left(3\right)}\right),
\end{equation}
and
\begin{equation}
\vec{F}\_{p-1}^{\left(2\right)}=\kappa\_{p}\left(\theta\_{p}-\theta\_{0}\right)\frac{1}{\sqrt{1-\left(\frac{\vec{r}\_{p-1,p}\cdot\vec{r}\_{p+1,p}}{\left|\vec{r}\_{p-1,p}\right|\left|\vec{r}\_{p+1,p}\right|}\right)^{2}}}\left[\frac{\vec{r}\_{p+1,p}}{\left|\vec{r}\_{p-1,p}\right|\left|\vec{r}\_{p+1,p}\right|}-\frac{\left(\vec{r}\_{p-1,p}\cdot\vec{r}\_{p+1,p}\right)\left|\vec{r}\_{p+1,p}\right|\hat{\vec{r}}\_{p-1,p}}{\left|\vec{r}\_{p-1,p}\right|^{2}\left|\vec{r}\_{p+1,p}\right|^{2}}\right]
\end{equation}
and
\begin{equation}
\vec{F}\_{p+1}^{\left(3\right)}=\kappa\_{p}\left(\theta\_{p}-\theta\_{0}\right)\frac{1}{\sqrt{1-\left(\frac{\vec{r}\_{p-1,p}\cdot\vec{r}\_{p+1,p}}{\left|\vec{r}\_{p-1,p}\right|\left|\vec{r}\_{p+1,p}\right|}\right)^{2}}}\left[\frac{\vec{r}\_{p-1,p}}{\left|\vec{r}\_{p-1,p}\right|\left|\vec{r}\_{p+1,p}\right|}-\frac{\left(\vec{r}\_{p-1,p}\cdot\vec{r}\_{p+1,p}\right)\left|\vec{r}\_{p-1,p}\right|\hat{\vec{r}}\_{p+1,p}}{\left|\vec{r}\_{p-1,p}\right|^{2}\left|\vec{r}\_{p+1,p}\right|^{2}}\right].
\end{equation}

## Equations of motion

Given that typical epithelial cell sizes are in the micron range with cell speeds of the order of a few $\mu m/s$, inertial effects do not
affect cell dynamics and it is common use overdamped equations of motion to describe cell motion. This is a first order ordinary differential equation
which is just a balance between the frictional force $\vec F^{fr}\_i = -\gamma \vec v\_i$ (where $\gamma$ is the friction coefficient and
$\vec v\_i=\dot{\vec r}\_i\equiv\frac{d\vec r\_i}{dt}$ is the velocity of particle $i$) and the forces acting on the cell. The main novelty of the SPV and AVM
approaches is the introduction of the active force in addition to the external and internal (by surrounding cells) forces. This force is designed to mimic
cells ability to move on its own. The molecular machinery responsible for cell crawling is very complex and not fully understood. For simplicity, within the
AVM we assume that each cells receives an active force of strength $\alpha$ in the direction of vector $\vec n\_i$. $\vec n\_i$ is the "polarity" vector
that determines cell directionality, i.e., a very crude approximation of direction of cell motion. SAMoS implements several models for polarity alignment
for cells, either to some external cue or to the neighbouring cells.

Therefore, the equations of motion for cell centres and polarity vectors are
\begin{eqnarray}
\gamma \frac{d\vec r\_i}{dt} &=& \alpha\vec n\_i + \vec F\_i + \vec \nu\_i(t)\\
\gamma\_r \frac{d\vec n\_i}{dt} &=& \vec \tau\_i + \vec \nu^r\_i(t),
\end{eqnarray}
Where force $\vec F\_i$ is given in Eq. (\ref{eq:avm\_force}), $\vec \nu\_i$ is the positional noise (typically, but not necessarily thermal in nature),
$\vec\tau\_i$ is the alignment torque (may be absent in the simplest cases) and $\vec \nu^r\_i$ is the rotational noise with similar properties
as the positional noise.

## Cell division and death

In order to mimic actual biological processes, cells need to be allowed to grow, divide and die. Currently, SAMoS supports simple growth, i.e. in each
time step cell's are increases by a factor
\begin{equation}
A\_i(t+dt) = (1+fdt)A\_i(t),
\end{equation}
where $dt$ is the time step, $f$ is the growth rate per unit time and $A\_i(t)$ and $A\_i(t+dt)$ is the area of cell $i$ at times $t$ and $t+dt$,
respectively. In each step cell "age" is incremented.

There are many different ways to model cell division. We use the simplest one in which cell is set to divide once it reaches a critical area $A\_c$. If the
cell area $A\_i$ exceeds $A\_c$, probability of division grows linearly with $A\_i - A\_c$. Once the cell is set to divide, two daughter cells are created.
Those cells are places along the direction of the director vector at a distance $\alpha$. Ages of two daughter cells is set to zero.

Similarly to the cell division, there are many ways one can model cell death. We opt for a simplest possible one, where the cell dies after it reaches certain
"age"

**NOTE!**

Implementing more complex division and death processes into SAMoS is straightforward. Please e-mail the 
lead developer for the instruction on how to do it.

## Comparison with SPV

The SPV mode uses aa slightly different expression for the energy. Namely, the SPV uses
\begin{equation}
E\_{SPV}=\sum\_{i=1}^{N\_{face}}\left[\frac{K\_{i}}{2}\left(A\_{i}-A\_{i}^{\left(0\right)}\right)^{2}+\frac{\Gamma\_{i}}{2}\left(P\_{i}-P\_{i}^{\left(0\right)}\right)^{2}\right],
\end{equation}
with $P\_{i}^{\left(0\right)}$ being the preferred perimeter of cell $i$, akin to the native area $A\_{i}^{\left(0\right)}$. We can rewrite
this as,
\begin{equation}
E\_{SPV}=\sum\_{i=1}^{N\_{face}}\left[\frac{K\_{i}}{2}\left(A\_{i}-A\_{i}^{0}\right)^{2}+\frac{\Gamma\_{i}}{2}P\_{i}^{2}-\Gamma\_{i}P\_{i}^{0}P\_{i}+\frac{\Gamma\_{i}}{2}\left(P\_{i}^{0}\right)^{2}\right].
\end{equation}
We can readily drop the last term as it does not contribute to the force. If for a moment we assume that $\Gamma\_{i}$, $P\_{i}^{\left(0\right)}$
and $K\_{i}$ do not depend on the cell $i$, we can rewrite the last expression as
\begin{equation}
E\_{SPV}=\frac{K}{2}\sum\_{i=1}^{N\_{face}}\left(A\_{i}-A\_{i}^{0}\right)^{2}+\frac{\Gamma}{2}\sum\_{i=1}^{N\_{face}}P\_{i}^{2}-\Gamma P^{\left(0\right)}\sum\_{i=1}^{N\_{face}}P\_{i}.
\end{equation}
Furthermore, perimeter $P\_{i}=\sum\_{\nu\in l\_{i}}l\_{\nu,\nu+1}$ allowing us to write the last term as
\begin{equation}
-\Gamma P^{\left(0\right)}\sum\_{i=1}^{N\_{face}}\sum\_{\nu\in l\_{i}}l\_{\nu,\nu+1}.
\end{equation}
Comparing this expression to the last term in Eq. (\ref{eq:vertex\_model\_energy}) we read off
\begin{equation}
\lambda=-\Gamma P^{\left(0\right)},
\end{equation}
or
\begin{equation}
P^{\left(0\right)}=-\frac{\lambda}{\Gamma}.
\end{equation}
Bi, *et al.* identify dimensionless shape parameter $p\_{0}=\frac{P^{\left(0\right)}}{\sqrt{A^{\left(0\right)}}}$. In our description this parameter becomes,
\begin{equation}
p\_{0}=-\frac{\lambda}{\Gamma\sqrt{A^{\left(0\right)}}},
\end{equation}
indicating that $\lambda<0$.

# Examples

Examples are given complete with configuration file and input files in the tutorial/examples folder.
An explanation of how to generate a qualitatively similar input file is also given for each example.
To see more details about each example please read the configuration file carefully.

## Active cells with fixed boundary

An important consideration with the Active Vertex Model is stabilising the boundary. We start with the strictest boundary conditions.
In this case we place 496 immobile boundary particles in a dense line surrounding the cells. All cell voronoi centres interact with eachother
and with the boundary via soft repulsion. This soft interaction is set sufficiently strong that cells have no chance to break through the boundary wall.

**Fig. 6:**  The initial state and the state of the cell patch after 1000 timesteps. Some cells have exchanged neighbours
between the first and second images.

In this simulation cells have a shape parameter $p\_0$ of 3.22. They cannot intercalate freely, rather in order to change neighbours they
must be driven by a strong active force as is happening here. In this case the active force is a applied to each cell in a random direction
which changes over time according to a rotational noise. The boundary is entirely stable despite strong active forces directed towards it.

Input files: fixed.input, fixed.boundary   
Configuration: cells\_fixed.conf

Samos includes scripts for building input configurations which place particles randomly inside the boundary. In order to obtain
a highly regular arrangement like the one in Figure 6, first frame, we can give all particles a soft repulsive force and use samos to relax the system.
The **soft\_on\_sphere** example does this relaxation followed by the simulation in one configuration file. Here the steps are done separately.

```
# Produce the initial input file. The '-bp' (boundary packing) argument sets the density of boundary particles.
# [-a <area>, -n <No. cells>, -l <radius>]
$ python utils/pvmodel/build_epithilial.py -type Circle -l 30 -n 1000 -bp 4. -a 3.0 -o out.input
# This produces a file 'out.input'. We can obtain the boundary information immediately.
$ utils/pvmodel/constructmesh.py eb out.input 
# The file is named 'out.boundary'. For relaxing the system obtain relax.conf 
# and check that it is reading 'out.input', then run it with Samos.
# After 1000 timesteps 'relax_0000001000.dat' will contain the new particle positions but 
# this output file is not the same format as an input file.
$ mv relax_0000001000.dat fixed.input 
$ utils/pvmodel/constructmesh.py makeinput fixed.input 
# The constructmesh.py routine overwrites fixed.input and leaves fixed.input.save in case 
# something went wrong.
# Rename your boundary file to keep good track of which input it is paired with.
$ mv out.boundary fixed.boundary
```

**WARNING!**

- There are many routines in constructmesh.py for manipulating input configurations and this code is under heavy development as of 12/10/16.
  The behaviour of the constructmesh.py executable may change.
- The configuration **relax.conf** uses a brownian integrator by default. If Samos crashes due to heavily overlapping particles then switch
  to nve integrator.

## Growth and division

It is possible have a cell patch gradually grow and divide from a handleful of cells to tens of thousands in a few hours of computational time.
While cells grow gradually by incrementing their preferred areas, cell division in the AVM is an inherently discontinuous process whereby a particle
is removed and two new ones added at new locations mirrored in the division plane. In order to maintain the stability of the simulation it is useful to
phase in the forces due to the new cells gradually. A smaller timestep is an even more effective measure to improve stability. In this example
the shape index of the cells is set to 3.10 which corresponds to quite regular cell shapes. Cell divisions under these conditions bear a vanishing risk
of causing a simulation crash and the computational time required to simulate the exponentially growing number of cells becomes the limiting factor.
In this case the patch with 24083 cells after less then 12 hours of computation is shown. Adjusting growth rate and division rate parameters leads
to very different distributions of cell areas.

**Fig. 7:**  Patches are made up of 37, 124 and 24083 cells respectively. Cells have a chance to divide if their area is greater
than 2.8 after which the probabilty of a cell to divide increases linearly with its area.

Input files: sfinal.input, sfinal.boundary   
Configuration: growth.conf

To produce suitable initial conditions follow exactly the same steps as the previous example.

```
# Produce a tiny patch of 30 randomly positioned particles. 
$ python utils/pvmodel/build_epithilial.py -type Circle -l 5 -n 30 -bp 2.5 -a 3.0 -o out.input 
# Now follow steps from previous example.
```

## Multiple Cell Types

This example is constructed by randomly choosing 50% of the initial cells to be assigned a new type in the input file.
The lambda ($\Lambda$) parameter is then specified in the configuration file to be -6.3 except for edges formed by a pair
of cells of differing type in which case it is -5.8. The effect is that cell boundaries between the cell types are
energetically unfavourable. The system is then given a finite temperature and is allowed to settle into a
low energy state whereby cells of the same type are aggregated.

**Fig. 8:**  Here 500 cells of type A and 500 cells of type B, initialy randomly distributed, find a local energy minimum
where cells of the same type neighbour eachother.

Input files: epi.input, epi.boundary   
Configuration: sorting.conf

Using randomly oriented active forces instead of thermal noise will likewise lead to interesting sorting patterns.
The parameters $K$ and $\Gamma$ can be set for each cell type independently. Also lambda can be specified for every type of edge,
even the boundary edges.
The ability to specify parameters for different types of particles is a powerful tool.

```
# In this case choose a low boundary packing. We don't want a solid wall of boundary particles 
# but a flexible boundary instead.
$ python build_epithilial.py -type Circle -l 30 -n 1000 -bp 1.5 -a 3.0 -o out.input
# Follow same steps as the first example to produce the .boundary file and relax the system. 
# Then change the type of half of the cells from 2 to 3
$ utils/pvmodel/constructmesh.py sampletype out.input 2 3 0.5
```

# References

1. Dapeng Bi, X. Yang, M. C. Marchetti, M. L. Manning, *Motility-driven glass transitions in biological tissues*, Phys. Rev X **6** 021011 (2016).

SAMoS (c) 2013-2016, University of Dundee
